# Nanopore-Mediated Assembly Enables Precise and Continuous Synthesis of mRNA-Encapsulated Lipid Nanoparticles for COVID-19 Vaccines

**DOI:** 10.1021/acsnano.6c00729

**Published:** 2026-07-16

**Authors:** Zhixiang Liu, William Stewart, Yilong Teng, Yufeng Song, Maoping Tang, Yuxuan Guo, Xue-Qing Zhang, Kamalesh K. Sirkar, Xiaoyang Xu

**Affiliations:** † Department of Chemical and Materials Engineering, 5965 New Jersey Institute of Technology, Newark, New Jersey 07103, United States; ‡ Shanghai Frontiers Science Center of Drug Target Identification and Delivery, School of Pharmaceutical Sciences, National Key Laboratory of Innovative Immunotherapy, 12474 Shanghai Jiao Tong University, Shanghai 200240, P. R. China; § Department of Biomedical Engineering, New Jersey Institute of Technology, Newark, New Jersey 07103, United States

**Keywords:** lipid nanoparticles, mRNA vaccines, RNA therapeutics, hollow fiber membrane, continuous manufacturing

## Abstract

RNA therapeutics are reshaping modern medicine, as exemplified by the rapid deployment of lipid nanoparticle (LNP)-based mRNA vaccines during the COVID-19 pandemic. However, the ability to reproducibly generate LNPs with precisely defined physicochemical properties at scale remains a critical challenge, particularly as the particle size and size distribution strongly influence biodistribution, cellular uptake, and therapeutic efficacy. Conventional mixing technologies ensure reproducibility but offer limited control over interfacial mixing and nanoparticle assembly, constraining both tunability and scalability. We present a hollow fiber membrane (HFM)-based platform that leverages dense arrays of nanoscale pores to mediate uniform, highly localized interfacial mixing, enabling controlled lipid self-assembly and RNA encapsulation. This nanopore-mediated architecture allows continuous, high-throughput synthesis of LNPs with tunable particle sizes, narrow size distributions, and high encapsulation efficiencies, with particle characteristics directly correlated to the membrane pore size. HFM-derived LNPs exhibit robust in vitro transfection and potent in vivo immune responses and are compatible with multiple lipid chemistries and nucleic acid payloads, including mRNA vaccine constructs. Together, this work establishes HFM-based nanopore assembly as a versatile and scalable approach for producing well-defined LNPs, with direct relevance to the current and future mRNA vaccine and therapeutic development.

## Graphical abstract

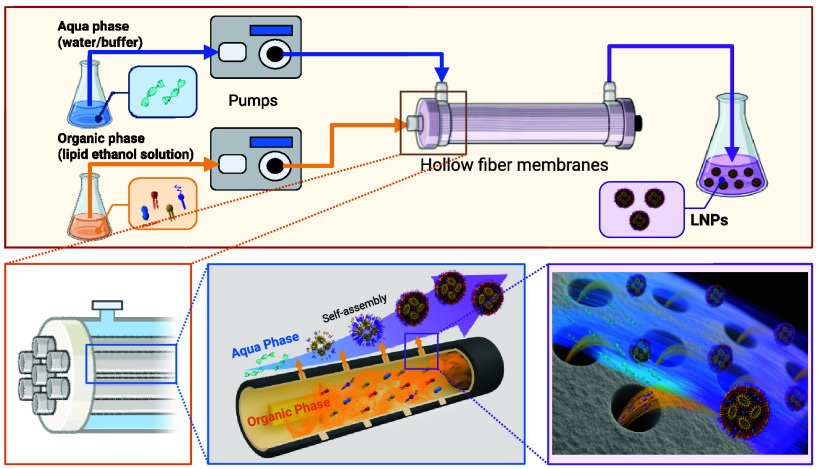

## Introduction

Lipid nanoparticles (LNPs) have become a foundational delivery platform for RNA therapeutics, driving breakthroughs in vaccines, gene therapies, and precision medicine.^1–3^ The rapid clinical deployment of COVID-19 mRNA vaccines validated LNP technology at unprecedented global scale, demonstrating not only safety and efficacy but also the feasibility of large-volume, reproducible nanoparticle manufacturing under stringent regulatory standards. This success has accelerated intense interest in extending mRNA-LNP platforms beyond prophylactic vaccination toward more complex therapeutic applications, including cancer immunotherapy and gene modulation, where delivery precision requirements are substantially higher. However, scalable and reproducible manufacturing of LNPs with tightly controlled physicochemical properties, including particle size, uniformity, and encapsulation efficiency, remains a critical challenge.^4–6^ Precise control over LNP size and size distribution is particularly important, as even modest variations can profoundly influence biodistribution, cellular uptake pathways, endosomal escape efficiency, and ultimately therapeutic efficacy and safety in vivo. Conventional microfluidic synthesis platforms, such as staggered herringbone mixers (SHMs), T-junctions, and confined impingement jet mixers (CIJM), rely on micrometer-scale channels (20–200 μm) to enhance mixing through vortex formation and chaotic advection.^7–9^ These designs require elevated Reynolds numbers (*Re* > 100) to induce turbulence-like flow and overcome diffusion limitations inherent in laminar regimes, which imposes a trade-off between mixing efficiency, shear stress, and scalability.^8,10,11^ Additionally, lithographic fabrication constraints, back-pressure buildup, and channel-to-channel variability complicate parallelization efforts, limiting throughput and manufacturing robustness.^12,13^ These intrinsic constraints highlight an urgent need for improved LNP synthesis methodologies that can bypass microfluidic mixing bottlenecks and achieve scalable, low-shear, and highly uniform nanoparticle formation.

Herein we report a nanopore-mediated lipid nanoparticle synthesis platform based on hollow fiber membranes (HFMs). This architecture features millions of uniformly sized nanometer-scale pores along the surface of a macroscale cylindrical fiber, transforming the mixing interface from a limited set of discrete microchannels into a massively parallel array of nanoscale nucleation sites.^14–17^ Moreover, individual fibers can be selected with defined pore diameters ranging from as low as 5 nm to submicron scales, enabling precision tuning of interfacial feature sizes to modulate mixing dynamics and control nucleation behavior.^18,19^ Hollow fiber membranes, widely used in industry, particularly in biomedical applications including filtration, dialysis, and purification, offer several intrinsic advantages, including scalable fabrication, tunable pore sizes, high surface area-to-volume ratios, and low transmembrane pressure differentials, all of which make them particularly well-suited for fluid processing at high throughput.^15,18,20^ Here, we repurpose this established platform for a distinct mechanistic role in controlled nanoparticle self-assembly. The organic lipid phase is radially injected through these nanopores into a coaxial aqueous stream flowing along the fiber exterior.^21,22^ Initially, droplets rapidly diffuse radially over millisecond time scales and are confined to characteristic lengths of approximately 60–80 μm per fiber.^14,21^ Following this rapid radial diffusion, the droplets are subsequently subjected to significant advective transport within the continuous aqueous phase, as indicated by the relatively high Peclet number greater than 10,000.^21,22^ Due to the radial nanodroplet formation the advective mixing occurs over greatly reduced axial mixing lengths (∼100 μm), more than 2 orders of magnitude shorter than staggered herringbone mixers, and over 3 orders of magnitude shorter than unstirred microchannels at similar Peclet numbers, ensuring efficient downstream dispersal of lipid material.^7,12,23^ This combinatorial mechanism establishes a continuous feed-and-fill mode wherein the ethanolic lipid phase is radially extruded through billions of nanopores, generating nanoscale droplets that instantly diffuse into the surrounding aqueous phase. These droplets are then swiftly entrained and advected along the fiber’s axial flow, orchestrating a seamless transition from radial diffusion to longitudinal convection. This highly coordinated interplay promotes uniform, spatially distributed mixing and synchronized nucleation across the full membrane surface, facilitating tight control over self-assembly kinetics under moderate Reynolds numbers and low-shear conditions. As a result, the system preserves RNA integrity while delivering exceptional throughput, particle uniformity, and formulation reproducibility.^2,24,25^

The nanoscale mixing advantages of the HFM platform are dramatically amplified by its densely packed nanopore array geometry. With an average porosity of 50%, the smallest commercially available modified polyether sulfone (mPES) HFM contains approximately 6.6 × 10^12^ discrete 10 nm pores per fiber.^14,15^ This creates a vast, parallelized field of rapid, simultaneous mixing zones with minimal hydraulic resistance and characteristically minimal mixing lengths.^20,21^ Compared to CIJM and microfluidic constructs, this represents multimagnitude differences in both the number of individual mixing sites and the reduced mixing lengths, yielding unparalleled mixing performance and uniformity of the LNPs.^8,9,11,26^ Computational fluid dynamics (CFD) simulations confirm that the HFM platform increases the effective interfacial area by over 5-fold compared to conventional microfluidic mixers, resulting in mixing efficiencies greater than 95%, while minimizing pressure gradients and eliminating velocity discontinuities inherent in parallel microchannels.^20,21,27–31^ Unlike SHM or impingement jet mixers, which face fundamental scaling and uniformity challenges, the HFM design already utilizes the modular bundling of fibers within cartridges, and cartridges can be easily parallelized to achieve continuous, high-throughput LNP production with tunable particle sizes, low polydispersity, and high encapsulation efficiency.^12,15,32^

Importantly, HFM-derived LNPs demonstrated potent in vitro transfection and in vivo immune responses equivalent or superior to benchmark formulations, confirming their therapeutic efficacy while showcasing broad compatibility across diverse lipid chemistries (ALC-0315, MC3, AA3-Dlin) and nucleic acid cargos, including mRNA, siRNA, and plasmid DNA. This performance underscores the modular, formulation-agnostic nature of the platform, which decouples LNP assembly from fixed microfluidic architectures and enables robust, tunable synthesis across a wide array of nanoparticle designs and therapeutic payloads. The hollow fiber membrane system introduces a fundamentally new framework for LNP production, replacing chaotic, shear-driven mixing in micrometer-scale channels with massively parallel, coupled diffusion-advection transport from billions of nanometer-scale pores. This shift grants unprecedented control over nucleation and self-assembly dynamics, drastically improving particle uniformity, preserving RNA integrity, and enabling continuous, high-throughput operation at an industrial scale. By overcoming entrenched limitations in shear stress, throughput, and reproducibility, the HFM platform establishes a transformative paradigm for nanoparticle preparation and nucleic acid encapsulation, one defined by precision, scalability, and universal applicability across the rapidly evolving landscape of nucleic acid therapeutics and clinical applications.^1,3,24,25^

## Results and Discussion

### High-Throughput Hollow Fiber Membrane Platform for Continuous LNP Production

A hollow fiber membrane (HFM) method was developed to establish a scalable platform for lipid nanoparticle (LNP) synthesis, in which the lipid–ethanol phase was driven through nanoscale membrane pores into a surrounding aqueous buffer containing nucleic acids (Figure 1). In this configuration, high-performance liquid chromatography (HPLC) pumps delivered the organic phase through the bore inlet and the aqueous buffer through the shell inlet, while the bore outlet was sealed to force radial diffusion of ethanol across the membrane (Figure 2A). This geometry generated numerous parallelized mixing sites along the entire fiber length, in contrast to the limited microscale junctions of conventional microfluidics, enabling highly efficient LNP self-assembly. Initial investigation into the effects of buffer species, molarity, pH, lipid-PEG concentration, and total lipid concentration on the size, polydispersity index (PDI), and encapsulation efficiency (EE%) of LNP’s produced via HFM method demonstrated that LNPs with tunable particle sizes between 75 and 250 nm were generated using the HFM method (Figure S1). Notably, only decreasing the buffer pH between 7.0 and 3.0 and increasing lipid-PEG concentration up to 3.0% resulted in appreciable reductions in particle sizes from ∼250 nm to ∼75 nm (Figure S1). Optimizing buffer composition significantly influences nanoparticle size, PDI, and EE%, underscoring the strong correlation between buffer conditions and the resulting physicochemical properties of LNPs (Figures S2 and S3).

**Figure 1:**
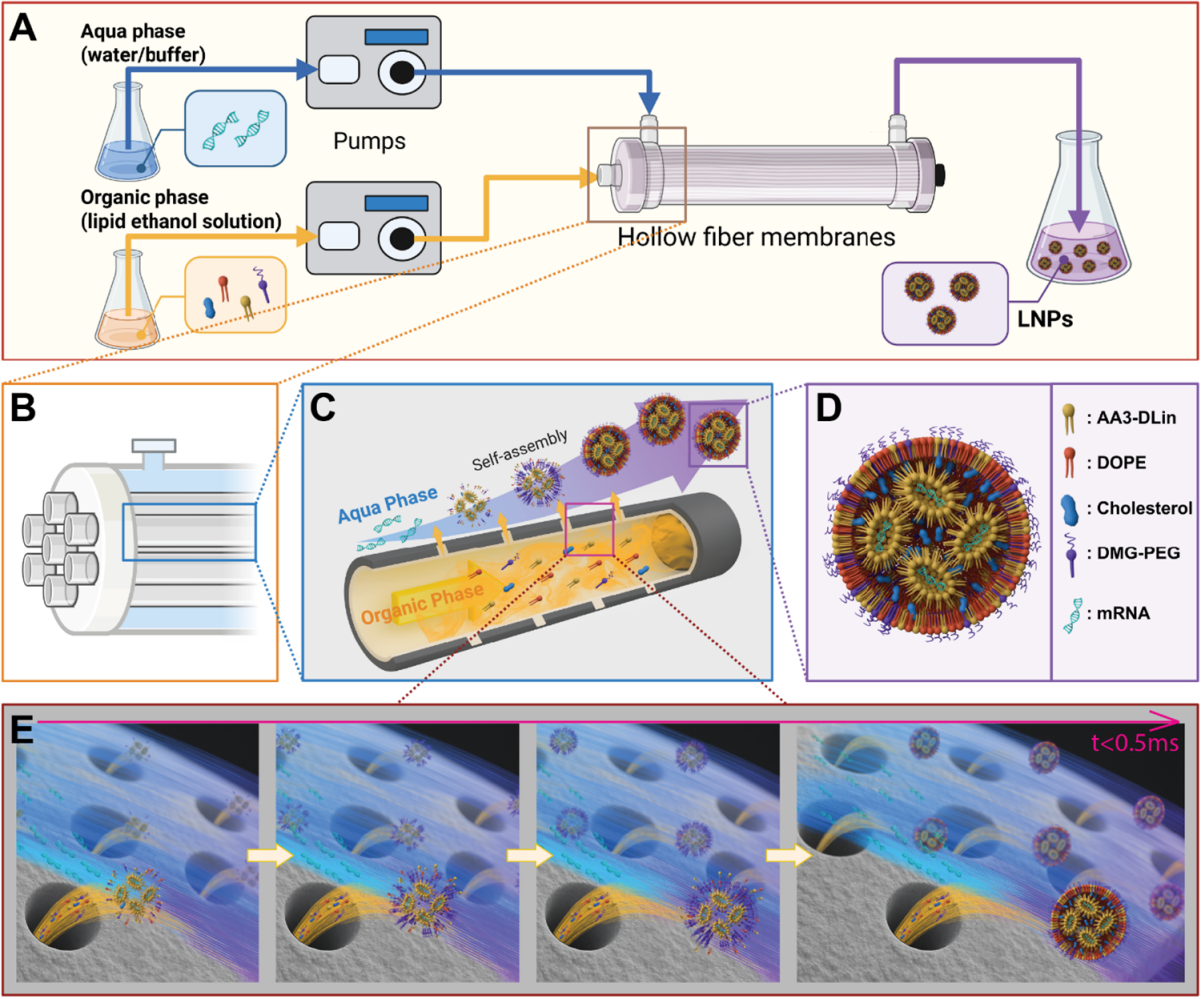
Schematic illustration of mRNA-encapsulated lipid nanoparticle synthesis via hollow fiber membrane. (A) Schematic illustration detailing pump configuration and sealed HFM bore outlet port to facilitate LNP synthesis via nanopore-mediated interfacial mixing. (B) Schematic illustration of the fibers in the HFM module. (C) Schematic illustration of the additive effect for LNP self-assembly via the densely porous HFM architecture. (D) 3D schematic illustration of the LNP. (E) Schematic illustration detailing transverse organic phase diffusion radially through a single HFM nanopore into the tangentially flowing aqueous phase and rapid subsequent advection and interfacial mixing to promote self-assembly of LNPs encapsulating nucleic acids.

**Figure 2:**
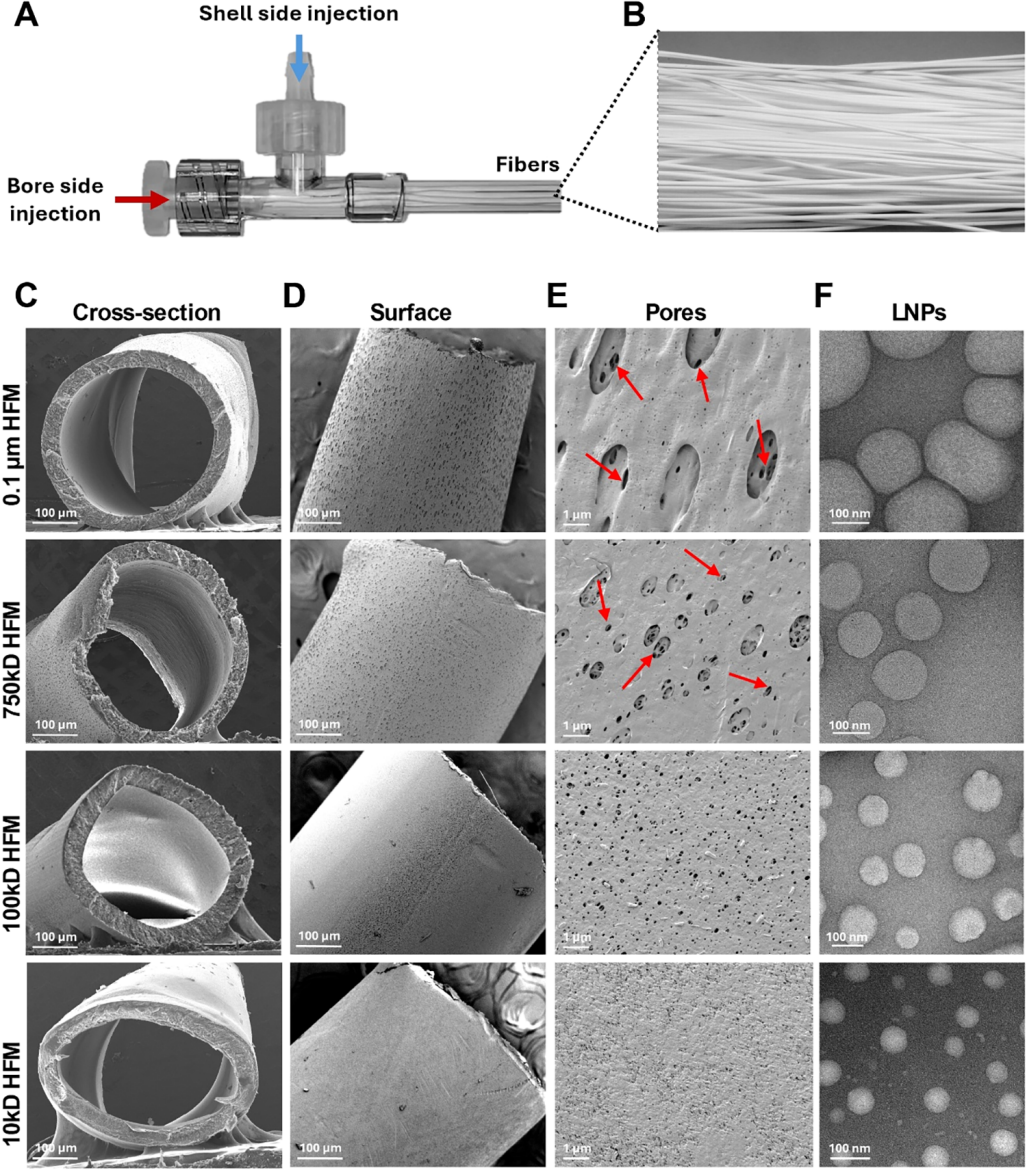
Characterization of the pore size of different MWCO hollow fiber membranes and the effect of pore size on the particle size and morphology of resultant LNPs produced via HFM. (A) Image of a HFM, and identification of the bore side for injection of organic phase into the fiber lumen, and shell side for injection of the continuous aqueous phase. Image of a network of multiple fibers bundled together in single HFM apparatus. (B) Image of network of multiple fibers bundled together in single HFM apparatus. (C) Cross-sectional SEM images of mPES HFM’s with MWCO sizes of 10 kDa, 100 kDa, 750 kDa, and 100 nm. Scale bar = 100 um. (D) SEM image of HFM surface of 10 kDa, 100 kDa, 750 kDa, and 100 nm mPES membranes. Scale bar = 100 um. (E) Increased magnification of SEM image of HFM surface of 10 kDa, 100 kDa, 750 kDa, and 100 nm mPES membranes to allow investigation of pore size differences based on MWCO values. Scale bar = 1 um. (F) TEM image of representative LNPs produced via HFM method and modulation of resultant particle size via reduced HFM pore size. Scale bar = 100 nm.

Geometrical parameters such as pore size, fiber length, and the number of fibers were easily adjusted through HFM cartridge and fiber selection, providing modular control over mixing efficiency and production capacity (Figure 2A,B). Differences in actual pore size for HFM’s with varied MWCO size cutoffs were verified by cross-sectional and surface Scanning Electron Microscope (SEM) analysis to reveal significantly smaller pores approximating 2.3 nm for 10 kDa MWCO HFM’s, and upward of 50 nm for 750 kDa MWCO HFMs (Figure 2C). Process parameters such as total flow rate (TFR) and flow rate ratio (FRR) were then precisely tuned by adjusting pump settings, offering straightforward control of particle characteristics. To further streamline manufacturing, we implemented a two-stage, in-series HFM process in which LNPs were synthesized in the first module and subsequently purified and buffer-exchanged by 8× diavolume cycling in a second module (Figure S11). LNP purification and ethanol concentration in the LNP solution were monitored via GC, with reduction of ethanol concentration below 2% after 8× diavolumes of buffer exchange, and approaching 0% after 12× diavolumes of buffer exchange (Figure S12). This continuous configuration eliminated the need for offline solvent removal and buffer exchange steps.

The scalability of the HFM platform was evaluated by varying the number of fibers per cartridge and parallelizing membrane modules. Increasing the number of fibers resulted in a corresponding increase in LNP production rate, from 15 mL/min using a 2-fiber HFM to 20 mL/min for 12 fibers and up to 80 mL/min for a 78-fiber configuration (Figure 4J,K). These results demonstrate that the production capacity can be systematically increased through module parallelization and fiber scaling. Rather than relying on increased flow velocity within a single channel, the HFM platform enables throughput enhancement through distributed nanoscale injection across a large number of pores. While direct comparison of absolute throughput with microfluidic systems depends on specific device design and operating conditions, these findings highlight the scalability potential of the HFM architecture. The combination of nanoscale mixing length and high pore density provides a distinct framework for controlling nanoparticle formation while allowing flexible adjustment of the production capacity.

### CFD Simulations Reveal Nanoscale Mixing Advantages of HFMs

To gain mechanistic insights into nanopore-mediated mixing in hollow fiber membranes (HFMs), computational simulations were performed using a simplified 2D pore-channel model that approximates the nanoporous architecture as an array of uniformly distributed injection sites (Figure 3A). The simulations are designed to capture the early-stage hydrodynamic interaction at the moment of contact between the injected organic stream and the surrounding aqueous flow, rather than to resolve detailed molecular transport or lipid self-assembly kinetics. Accordingly, the simulation results are interpreted qualitatively and used primarily for the comparative analysis of different pore size conditions. Transient simulations were conducted to visualize the spatial evolution of the injected stream and the formation of the interaction region during mixing (Figure 3B). In this context, the level set field is used as a numerical indicator of the transient organic-rich region, enabling quantitative characterization of the interaction area and its dependence on pore size.

**Figure 3:**
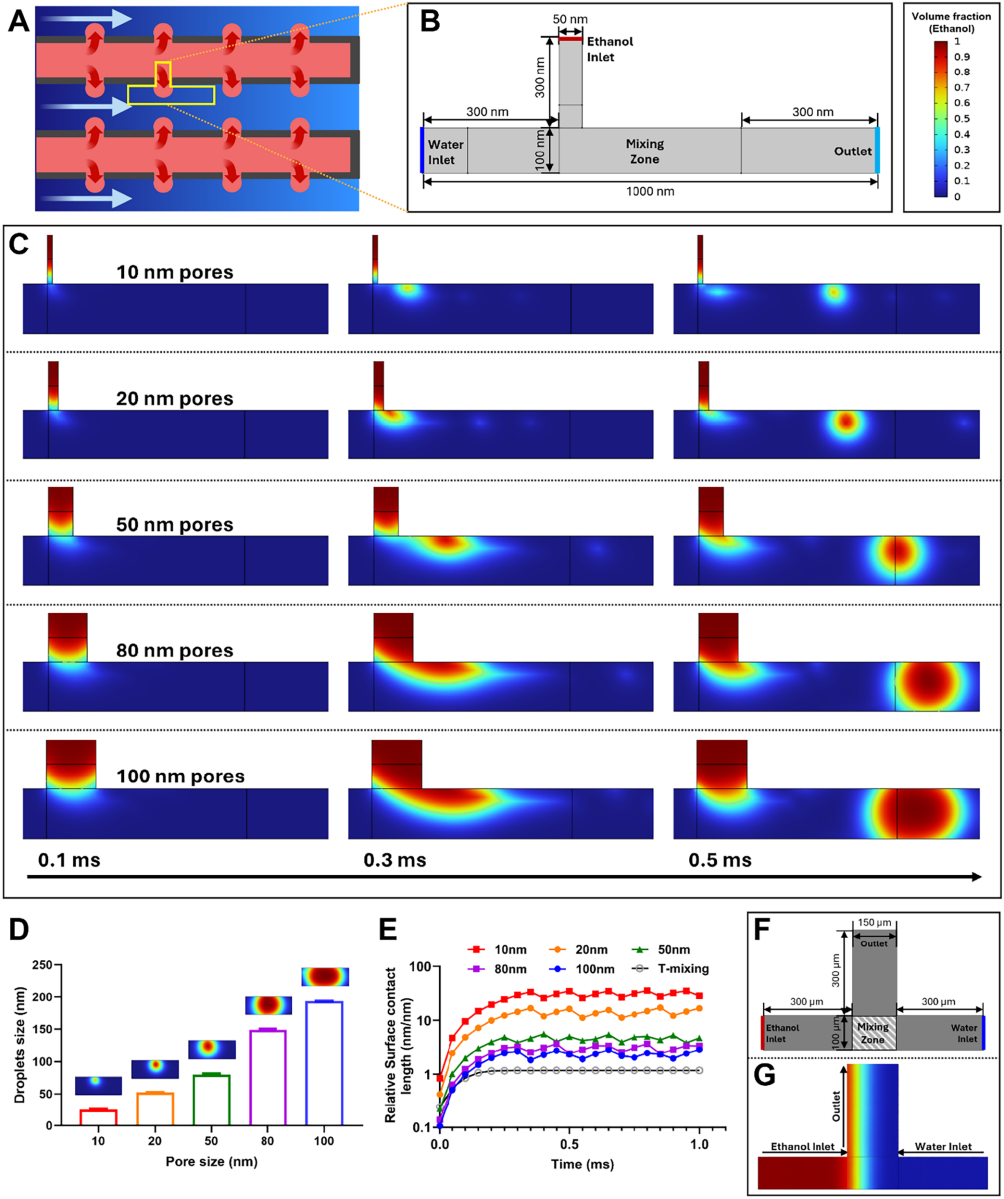
Computational flow dynamics simulation results for HFM LNP method, and comparison to microfluidic T-Junction mixing. (A) 2D schematic of nanopore-mediated HFM mixing and (B) 2D projection of a 50 nm mixing zone perpendicular to the surface of the fiber. (C) Simulated Level Set distributions illustrating the effect of pore size (10–100 nm) on the spatial distribution of the injected organic-rich region. (D) Characteristic dimension of the injected region as a function of pore size at 0.5 ms after injection. (E) Comparison of the relative contact surface generated within HFMs of different pore sizes and a conventional T-junction mixer. The interaction region was defined as the Level Set transition zone (0.4 < ϕ < 0.6), and the corresponding interaction area was calculated by surface integration and normalized by the total computational domain area to obtain a dimensionless relative contact surface. (F) Schematic of T-Junction micromixer simulation model utilizing 100 um wide inlet channels and 150 um wide outlet channel. (G) Simulated spatial distribution of the injected stream within the T-junction mixer used for qualitative comparison with the HFM architecture.

The simulation results reveal that decreasing pore size significantly alters the spatial distribution of the injected organic-rich region. For smaller pores (∼10 nm), the injected streams are more finely distributed and rapidly dispersed into the surrounding aqueous flow, resulting in a larger number of localized contact regions and reduced characteristic length scales of hydrodynamic interaction (Figure 3C).^15,21^ In contrast, larger pores (50–100 nm) produce coarser injected domains with less spatial dispersion, leading to more localized and less distributed interaction patterns. Consistent with this observation, the characteristic dimension of the injected phase domains decreases with pore size, showing an approximately 7.8-fold reduction as pore diameter decreases from 100 to 10 nm (Figure 3D). This trend indicates that nanopore size directly governs the initial spatial scale of the injected organic-rich region and its interaction with the surrounding flow field. To quantitatively characterize this behavior, the extent of hydrodynamic interaction between the injected organic-rich region and the surrounding flow was evaluated based on the Level Set field. Specifically, the interaction region was defined as the Level Set transition zone (0.4 < ϕ < 0.6), and the corresponding interaction area was obtained through surface integration and normalized by the total computational domain area to yield a dimensionless relative contact surface (Figure 3E). The results show that decreasing pore size significantly increases the spatial coverage of the interaction region. Smaller pores generate a higher density of distributed injection sites, resulting in a more extensive and spatially distributed interaction region. In contrast, the T-mixer lacks this radially injected parallelized nanoarray architecture and exhibits a substantially reduced relative contact surface at 1 ms and longer time scales.^7,23,28^ For comparison, published 3D T-mixer simulations were used as a reference, which showed similar ethanol-distribution patterns and provided qualitative support for the simulated flow behavior (Figure 3F,G).^28–30,33^

The nanoscale confinement and high density of injection sites collectively reduce the characteristic length scale associated with the injected organic-rich region and enhance the spatial uniformity of the distribution. This behavior is expected to increase the available interaction area and promote more homogeneous local mixing conditions during nanoparticle formation. Importantly, this effect arises from geometrically controlled flow distribution rather than turbulence, as the system operates in a viscous-dominated laminar regime.^14,21^ These results suggest that nanopore-mediated mixing fundamentally alters the spatial distribution of the injected stream, with smaller pores providing more favorable conditions for uniform nanoparticle formation.

### Tuning LNP Characteristics via HFM Physical Properties

Building on simulation insights, experimental investigation into the influence of membrane properties on LNP formation was performed by synthesis of LNPs using HFMs with varying molecular weight cutoffs (MWCO) and pore diameters, including 10 kDa (2–5 nm pores), 100 kDa (∼10 nm), 750 kDa (∼50 nm), and 0.1 μm pores (Figure 2A,B). Transmission electron microscopy (TEM) and SEM confirmed an inverse correlation between pore size and LNP diameter (Figure 2C–F). Pore density for the different MWCO HFM’s was determined by ImageJ analysis of the SEM images, with pore density increasing from 10.3 pores/100 um2 for the 750 kDa HFM to 132.1 pores/100 um2 for the 10 kDa HFM (Figure S10).

Dynamic light scattering (DLS) confirmed that smaller pores consistently yielded smaller, more uniform particles (Figure 4A–F). For instance, 10 kDa mPES membranes produced LNPs averaging 80–100 nm, whereas increasing the pore size to 750 kDa resulted in a particle size increase of 100 nm, and 0.1 μm membranes generated particles exceeding 250 nm. Although PDI increased slightly with pore size (ΔPDI < 0.1), all formulations remained well within pharmaceutical acceptability. Importantly, EE% was consistently high, greater than 80% across all membranes, demonstrating that RNA retention was not compromised by pore size. Analysis of the correlation coefficient between the pore size diameter of the HFM and resultant LNP physicochemical properties demonstrate that HFM pore size is positively correlated with LNP particle size resulting in an R2 value of 0.8233 (Figure S5). Further correlation analysis revealed pore size as the only parameter showing a positive and predictive relationship with LNP particle size, establishing it as a key determinant of nanoparticle dimensions (Figure S6).

**Figure 4:**
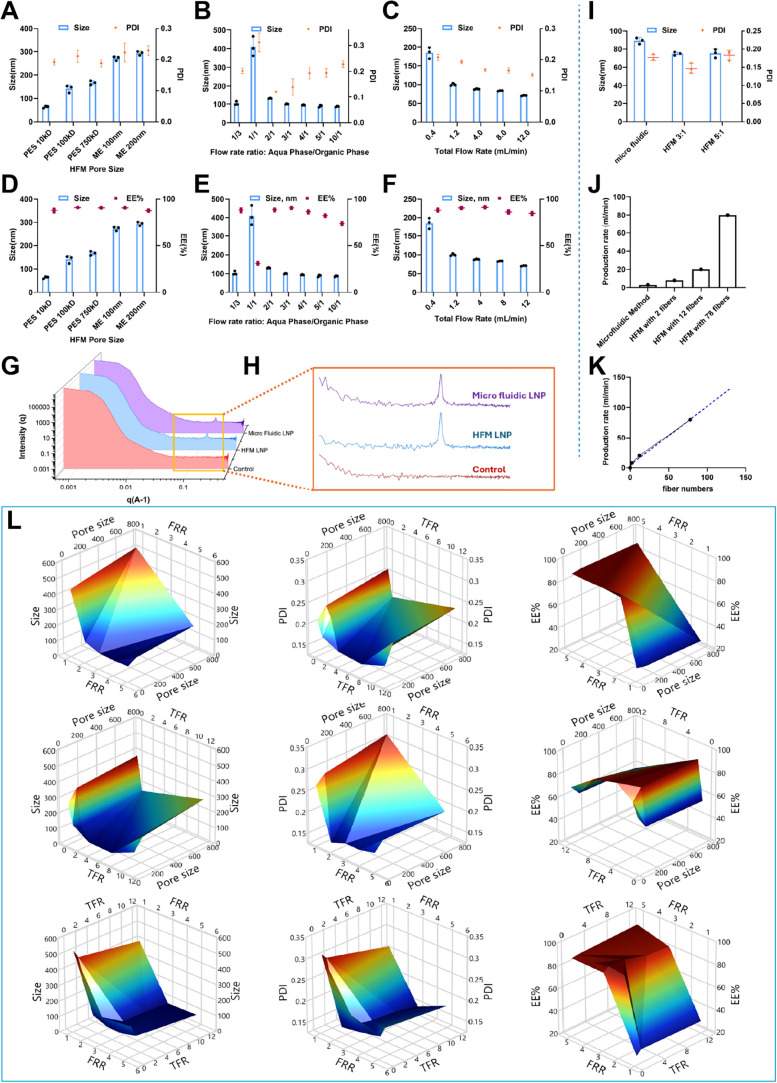
Influence of geometrical and process parameters on LNP characteristics synthesized via HFM method, and comparison of HFM method and microfluidic method. (A) Effect of HFM pore size on particle size and PDI of LNPs produced via HFM method. (B) Effect of FRR on particle size and PDI of LNPs produced via HFM method. (C) Effect of TFR on particle size and PDI of LNPs produced via HFM method. (D) Effect of HFM pore size on particle size and encapsulation efficiency of LNPs produced via HFM method. (E) Effect of FRR on particle size and encapsulation efficiency of LNPs produced via HFM method. (F) Effect of TFR on particle size and PDI of LNPs produced via HFM method. (G, H) Comparison of SAXS measurements of LNPs at different production methods (microfluidic LNP: size 85.8 nm, PDI 0.142, EE > 90%. HFM LNP: size 75.2 nm, PDI 0.135, EE > 90%). (I) Comparison of particle size and PDI of LNPs produced via microfluidic and HFM methods using equivalent process conditions. (J) Comparison of LNPs production rate via microfluidic method and HFM method with increasing number of fibers and (K) predicted production rate. (L) Critical factor determination and surface response analysis of the effects of HFM pore size, FRR, and TFR on the particle size, PDI, and encapsulation efficiency of LNPs produced via HFM method. *n* = 3 (microfluidic LNP: LNPs prepared via microfluidic mixer; HFM LNP: LNPs prepared via HFM platform).

Collectively, these findings position the HFM pore size as a direct, tunable geometric control lever for modulating LNP size and uniformity at the point of nucleation, enabling precise, reproducible, and scalable nanomanufacturing.

### DoE-Guided Tuning of Process Parameters for LNP Optimization

Following the identification of the effects of the HFM’s geometrical properties on LNPs, a Design of Experiments (DoE) framework was employed to systematically evaluate the impact of key chemical and process variables on LNP characteristics. Flow rate ratio (FRR) governs ethanol dilution rate and supersaturation-driven nucleation. Total flow rate (TFR) controls mixing time scales and shear-enhanced diffusion. Membrane pore size regulates nanoscale injection velocity and interfacial area density. The experimental results for each group are based on three independent experimental batches.

FRR emerged as the dominant process-related factor controlling particle size and encapsulation efficiency (EE%). Increasing the aqueous-to-organic ratio from 1:1 to 3:1 reduced LNP diameter and elevated EE% to ∼85%. Beyond an FRR of 3:1, further increases did not confer additional size reduction; instead, polydispersity index (PDI) increased and EE% declined toward 70% (Figure 4B,E). At low FRR, the system may experience insufficient ethanol dilution rate, leading to delayed supersaturation. This can result in heterogeneous nucleation and broader size distribution, reflected in variations in size, PDI, and EE. TFR also modulated nanoparticle formation by influencing mixing efficiency. Raising TFR from 0.4 to 4 mL/min progressively decreased particle size and PDI, but improvements plateaued beyond 8 mL/min, suggesting saturation of mixing dynamics (Figure 4C,F). In contrast to FRR, TFR had minimal impact on EE%, which remained consistently high.

The DoE model further revealed synergistic interactions between the geometrical parameters (pore size) and process parameters (FRR, TFR), where low FRR combined with large pores produced the largest and least uniform nanoparticles, and higher FRR and TFR combined with smaller pores produced the smallest and most monodisperse LNPs (Figure 4L). Surface response analysis of these critical factors that impact particle size demonstrated that FRR, TFR, and pore size all had significant p-values less than 0.05. Surface response analysis identified TFR as the only parameter with a significant effect on both PDI and EE%, while pore size and FRR showed no measurable impact (Figure S4). Complementary least-squares analysis provided further insight into these correlations (Figures S7–S9).

Based on these findings, optimal conditions were established as FRR = 3:1 and TFR = 8 mL/min, producing ∼58 nm LNPs with low PDI and high EE%. These parameters were applied in all subsequent comparative studies and biological evaluations, ensuring robust, reproducible nanoparticle synthesis. This combination defines a robust, scalable operating window for precision nanomanufacturing of RNA-loaded LNPs, in which pore-mediated nucleation is optimally matched to solvent exchange and mixing kinetics.

### Comparison of HFM vs Microfluidics: Enhanced Control and Scalable Production

Direct benchmarking against conventional microfluidic mixers highlights the distinct advantages of the HFM platform. Across all tested FRRs, LNPs produced via HFMs were consistently smaller and exhibited lower PDI compared with microfluidic-synthesized particles, as determined by DLS (Figure 4I). Small-angle X-ray scattering (SAXS) analysis confirmed that the internal nanostructure of LNPs was preserved regardless of the production method, demonstrating that enhanced size uniformity did not compromise the supramolecular architecture (Figure 4G,H).^24,34^

In addition, capillary electrophoresis (CE) analysis confirmed that the integrity of the encapsulated mRNA was preserved during HFM processing. The mRNA integrity of LNPs prepared by microfluidic and HFM methods was 81.2% and 82.6%, respectively, with no statistically significant difference observed between the two groups (Figure S19). These results indicate that the nanopore-mediated mixing process does not induce detectable mRNA degradation and preserves cargo quality comparable to that of conventional microfluidic approaches.

LNPs prepared using the HFM platform demonstrated excellent physicochemical stability under both refrigerated and frozen storage conditions. Under refrigerated conditions (4 °C), the LNPs exhibited a slight increase in particle size over time, from 75 nm (Day 0) to 83 nm (Day 14), while PDI remained stable within a narrow range (0.12–0.15). Encapsulation efficiency was well maintained, remaining above 85% throughout the storage period. For frozen storage at −20 °C with 10% sucrose, the LNPs showed minimal changes after thawing, with no significant aggregation observed. Particle size remained largely unchanged (around 76 nm), and encapsulation efficiency was stable at approximately 87%. Furthermore, this LNP demonstrated robust tolerance to freeze–thaw stress. Under a single freeze–thaw cycle, neither the particle size nor the PDI exhibited any significant changes. Even after undergoing multiple freeze–thaw cycles, the particle size and PDI showed only a slight increase (from 75 to 85 nm, PDI from 0.12 to 0.16), while the reduction in encapsulation efficiency remained negligible (Figure S20).

Scalability further reinforced the superiority of the HFMs. Expanding the number of fibers from 2 to 12, and ultimately to 78, resulted in proportional linear increases in LNP yield without any deterioration in particle quality (Figure 4J,K). In contrast, microfluidic platforms are inherently limited by discrete channel numbers, constraining the throughput.^12^

Collectively, these results establish HFMs as a highly tunable and scalable platform, combining precise nanoparticle control with production rates suitable for both laboratory-scale studies and industrial-scale RNA-LNP manufacturing.

### Robust In Vitro Transfection and In Vivo Immunogenicity of HFM-LNPs

The transfection efficiency of HFM-derived LNPs was evaluated in HEK293 cells using EGFP mRNA. At 48 h post-transfection, ∼91.8% of cells were GFP-positive, slightly underperforming microfluidic LNPs (∼92.5%), with comparable mean fluorescence intensity (Figure 5A–C). Lipid composition further modulated efficacy: AA3-DLin LNPs, synthesized using the ionizable cationic lipid shown in Figure S13, achieved 91.8% GFP+ cells, surpassing MC3 (88.1%) and ALC-0315 (90.3%) formulations (Figure 5D–F). This demonstrates that the HFM platform effectively supported the synthesis of diverse LNP formulations, with AA3-DLin, ALC-0315, and MC3 compositions all yielding strong and comparable protein expression. Co-delivery of GFP and mCherry mRNAs demonstrated high dual-transfection efficiency (≥80% for both reporters; 88% GFP+, 97% mCherry+), confirming that HFM-LNPs support robust, sequence-independent mRNA delivery (Figures 5G–K and S14–S18). Biodistribution studies demonstrate that the physicochemical properties exhibited by LNPs prepared using HFM technology are highly consistent with those of established formulations whose biodistribution characteristics have been fully characterized (Figure S21).

**Figure 5:**
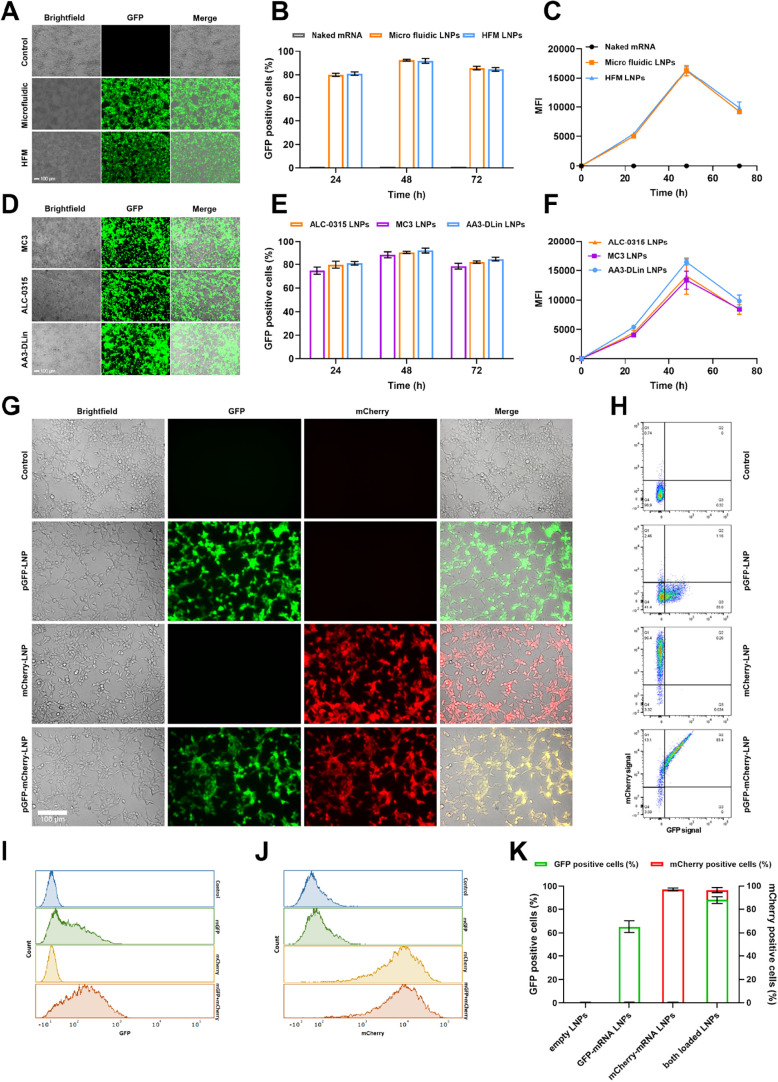
Comprehensive evaluation of AA3-DLin LNPs through the HFM method. (A) Fluorescence images of GFP-positive HEK293 cells transfected by AA3-DLin LNPs at 24 h with different methods. (B) Summary of GFP-expressing cell population transfected by different methods. (C) MFI of GFP-expressing cells based on the flow cytometry results. (D) Fluorescence images of GFP-positive HEK293 cells transfected by LNPs at 24 h with different LNP formulation. (E) Summary of GFP-expressing cell population transfected by different LNP formulations and (F) MFI of GFP-expressing cells. (G) Fluorescence images of GFP-positive and mCherry-positive HEK293 cells transfected by AA3-DLin LNPs at 24 h encapsulated with different mRNA. (H) Flow cytometry results and count summary with GFP (I) and mCherry (J). (K) Summary of GFP-expressing and mCherry-expressing cell population transfected by HFM-LNPs with different mRNA (scale bar = 100 μm).

In vivo immunogenicity was assessed in BALB/c mice following intramuscular vaccination with AA3-DLin spike mRNA LNPs (2 μg or 10 μg), prepared using either HFM or microfluidics (Figure 6A). Particle size analysis of AA3-Dlin LNPs produced by both the microfluidic and HFM methods was confirmed to be similar at 78.8 and 68.0 nm, respectively (Figure 6C). LNP surface morphology was confirmed to be spherical by Cyro-TEM (Figure 6C,D). Prime immunization elicited modest antibody titers (GMT: 808–1550), while booster dosing increased titers over 20-fold GMT: 19,118 for HFM vs 19,185 for microfluidic (Figure 6B). Cellular immune responses were similarly strong: both HFM and microfluidic LNPs induced elevated populations of IFN-γ+, IL-2+, and TNF-α+ CD4+ and CD8+ T cells, as confirmed by intracellular cytokine staining and ELISpot assays (Figure 6E–L). The absence of detectable IL-4 indicates a Th1-skewed immune profile. These results establish that HFM-derived LNPs achieve transfection and immunogenic performance equal to or exceeding microfluidic counterparts, while maintaining the added benefits of scalable, high-throughput production.

**Figure 6:**
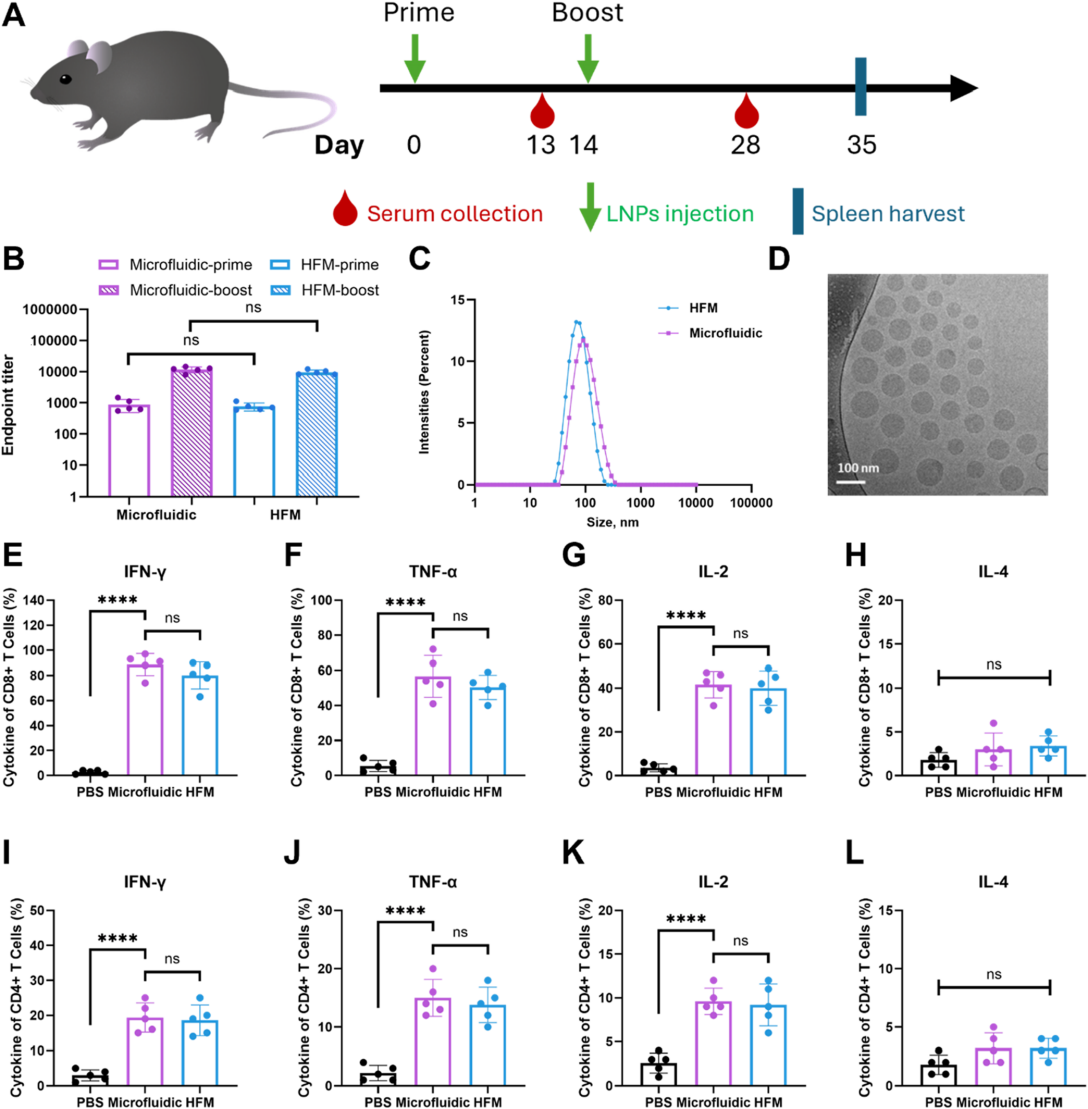
Immunogenicity of AA3-DLin COVID-19 vaccines. (A) Schematic illustration of mice immunization experimental design. (B) AA3-DLin vaccines produced by microfluidic and HFM methods induced robust SARS-CoV-2 spike-specific antibody end point titers in the immunized mice. (C) Particle size of AA3-Dlin LNP’s can be effectively tuned to match the particle sizes produced via the microfluidic method. (D) Cryo-TEM demonstrates uniform particle size and morphology of LNP’s produced via HFM. Scale bar = 100 nm. (E-H) CD8^+^ T cell production of IFN-γ, TNF-α, IL-2, and IL-4 after vaccination. (I-L) CD4^+^ T cells production of IFN-γ, TNF-α, IL-2, and IL-4 after vaccination. Vaccination was evaluated using HFM LNP and microfluidic LNPs, *n* = 5. All LNPs were encapsulated with 10 μg spike-encoded mRNA, and serum was collected after the second booster injection on day 28. Data are displayed as mean ± SD. A one-way ANOVA with multiple comparison tests and unpaired *t* test for comparison of two groups were used to analyze the statistical significance (ns, not significant; *p < 0.05, **p < 0.01, ***p < 0.001, ****p < 0.0001).

## Conclusion

The rapid expansion of RNA-based therapeutics, including mRNA vaccines, gene editing tools, and personalized precision medicine treatments, has placed unprecedented demands on lipid nanoparticle (LNP) manufacturing platforms, which are the most clinically advanced delivery systems for mRNA-based therapeutics.^1–3^ The global deployment of COVID-19 mRNA vaccines underscored both the power of LNP-enabled RNA medicines and the critical importance of scalable, reproducible nanoparticle manufacturing capable of meeting worldwide demand under strict quality constraints. Microfluidic systems such as T-junctions, staggered herringbone mixers (SHMs), and confined impingement jet mixers (CIJMs) have been widely adopted in early-stage production due to their ability to achieve well-characterized mixing regimes and reproducible nanoparticle size distributions.^7,9,11,23^ These systems rely on microscale channel features, typically 20–200 μm in width, where chaotic advection and controlled turbulence facilitate rapid micromixing and LNP nucleation.^7,8^ However, these microfluidic approaches are fundamentally constrained by their geometries and flow regimes. The need to operate at elevated Reynolds numbers (*Re* > 100) to sustain turbulent mixing introduces shear stresses that may compromise RNA integrity and limits operational flexibility.^2,6^ Moreover, microchannel architectures are prone to fouling, clogging, and high backpressures that restrict their translation to industrial settings.^12,18^ They also exhibit scaling limitations due to lithographic size limits, and face challenges with parallelization that often result in flow instabilities and interchannel variability.^12,13^ As a result, despite their utility in early product development, conventional microfluidic mixing platforms remain challenged by risk of shear-related degradation, susceptibility to fouling, and scale-up constraints, leaving a clear need for alternative manufacturing strategies capable of combining precision control with industrial scalability.^4,5^

Herein, we report a hollow fiber membrane pore-mediated method representing a transformative advance in lipid nanoparticle synthesis that exploits nanoscale transmembrane injection to achieve massively parallelized mixing and controlled nanoprecipitation. In this strategy, the lipid phase is driven through billions of nanometer-scale pores into a tangentially flowing aqueous buffer, generating nanodroplets that rapidly diffuse radially through the membrane and are chaotically advected into the aqueous flow regime, creating dense, localized interfacial mixing zones (Figure 1). HFMs, which are a mature and industrially established technology with well-established applications in ultrafiltration and dialysis, feature a densely porous nanostructured architecture comprising millions to trillions of pores per fiber, enabling a fundamental transformation of the mixing interface from discrete microchannels to a continuous, three-dimensional porous surface (Figure 2A–D).^14,15,20^ For instance, with porosity values approaching 50%, a single 20 cm mPES fiber with 10 nm pores contains approximately 6.6 × 10^12^ pores, each acting as an independent nanoscale mixing site. This massive increase in interfacial surface area provides both orders of magnitude greater mixing site density, as well as orders of magnitude reduced interfacial mixing feature size compared to lithographically fabricated microfluidic chips and CIJMs.^8,9,11,27^ Lipid phase injection through these nanopores into an aqueous buffer flowing tangentially outside the fiber lumen creates localized mixing zones characterized by intense microscale shear and rapid solute exchange.^14,22^ This confined, radial-to-axial nanoprecipitation mechanism is conceptually analogous to a large ensemble of CIJMs, where each pore injects a high-velocity nanodroplet of lipid solution into the aqueous phase. Unlike macroscale CIJMs, the HFM system achieves mixing at both the nanometer scale and under massively parallelized conditions, resulting in a unique hydrodynamic and mixing phenomena.

CFD simulations further support the experimentally observed trends and provide qualitative insight into nanopore-mediated flow behavior.^20,21,27,28^ A simplified two-dimensional model was employed to visualize the early-stage hydrodynamic interaction between transmembrane organic injection and the surrounding aqueous flow (Figure 3A,B). The simulations indicate that decreasing pore size leads to a more finely distributed injection of the organic stream into the aqueous phase, resulting in reduced characteristic length scales of hydrodynamic interaction and a more spatially distributed interaction pattern.^14,21^ In particular, smaller pores (∼10 nm) generate a dense array of localized injection points, producing a more homogeneous distribution of the injected stream compared to larger pores (Figure 3C–E). In particular, smaller pores (∼10 nm) generate a dense array of localized injection points, producing a more homogeneous distribution of the injected stream compared to larger pores (Figure 3C–E). In contrast, larger pores yield more concentrated injection structures with limited spatial dispersion, leading to less uniform hydrodynamic interaction. This nanopore-mediated injection mechanism differs fundamentally from conventional T-mixing configurations, where interaction is typically confined to a limited number of macroscopic contact regions. In the HFM system, the presence of a large number of nanoscale pores enables parallelized radial injection, resulting in a spatially distributed interaction process even under laminar, viscous-dominated flow conditions. Although the present model employs a Level Set formulation that does not explicitly resolve molecular diffusion, species transport, or lipid self-assembly in a fully miscible system, it provides a useful hydrodynamic approximation for visualizing the spatial distribution of the injected organic-rich region. Accordingly, the simulations are interpreted qualitatively and semiquantitatively, with emphasis placed on the relative influence of pore size on early-stage flow structure and interaction area. From a mechanistic perspective, the increased interaction area and more homogeneous spatial distribution generated by smaller pores are consistent with the experimentally observed reductions in particle size and dispersity. A larger number of distributed injection sites is expected to create a more uniform local environment for nanoparticle formation, thereby reducing spatial heterogeneity during the early stages of assembly. While the present model does not explicitly simulate nucleation, growth, or solvent exchange kinetics, the observed hydrodynamic trends support the hypothesis that nanoscale confinement and distributed injection contribute to improved control over nanoparticle formation. Therefore, the improved particle size uniformity observed experimentally can be associated with the nanoscale confinement and distributed injection enabled by the HFM architecture.

The effect of key geometrical and process parameters on resultant LNP properties was rigorously assessed through full-factorial Design of Experiments (DoE) and supported by CFD modeling.^35^ The primary determinant of LNP characteristics in the HFM-based system is the geometrical architecture of the hollow fiber membrane, particularly pore size, which directly modulates the spatial distribution of injected streams and the local interaction environment during particle formation. Modified poly­(ether sulfone) (mPES) membranes with molecular weight cutoffs (MWCOs) of 10, 100, and 750 kDa, corresponding to effective pore diameters of approximately 5–10 nm, ∼20 nm, and 50 nm, respectively, were systematically evaluated alongside mixed-ester (ME) membranes featuring larger pores (∼0.1 μm) (Figure 2A–C). Both electron microscopy (Figure 2D,E) and particle size distribution analysis revealed a clear correlation between pore size and resultant LNP diameter, where smaller pores consistently produced smaller and more monodisperse particles, with PDI remaining <0.2 across all conditions (Figure 2F). Despite variations in pore architecture, encapsulation efficiency (EE%) remained consistently high, indicating that the nanopore-mediated mixing mechanism effectively condenses RNA within ionizable lipid systems, independent of the pore architecture. Collectively, these findings position HFM pore size as a direct, tunable geometric control lever for modulating LNP size and uniformity at the point of nucleation, enabling precise, reproducible, and scalable nanomanufacturing.

Complementing the geometric control afforded by HFM pore size, our design of experiments (DoE) analysis identified flow rate ratio (FRR) and total flow rate (TFR) as critical process parameters for tuning LNP size, polydispersity, and encapsulation efficiency (Figure 4L). Across the tested design space, FRR exhibited a nonlinear relationship with particle properties, with an optimal value of ∼3:1 yielding the smallest, most monodisperse particles and highest EE% (Figure 4A–F). TFR primarily influenced size and PDI through shear-enhanced micromixing, with diminishing returns beyond ∼8 mL/min and minimal impact on EE%. Importantly, these effects were further enhanced by membrane geometry: 10 kDa MWCO HFM cartridges containing two 10 nm-pore fibers, operated at an FRR of 3:1 and TFR of 8 mL/min, consistently produced sub-50 nm LNPs with low PDI and greater than 80% encapsulation efficiency. This combination defines a robust, scalable operating window for precision nanomanufacturing of RNA-loaded LNPs, in which pore-mediated nucleation is optimally matched to solvent exchange and mixing kinetics.

The HFM platform was benchmarked against commercial microfluidic LNP synthesis systems across both production metrics and nanoparticle qualities. At identical FRRs and TFRs, LNPs produced via the HFM consistently exhibited smaller hydrodynamic diameters and lower PDIs than those generated by microfluidic devices as determined by dynamic light scattering (DLS). Transmission electron microscopy (TEM) was employed to characterize both the hollow fiber membranes and the resultant LNPs. TEM imaging of mPES fibers confirmed the uniformity of pore size distributions across the membrane surface, while TEM analysis of LNPs revealed spherical, well-defined particles in both HFM- and microfluidic-derived samples with no evidence of aggregation or morphological defects (Figure 2F). Small-angle X-ray scattering (SAXS) further demonstrated that the internal nanostructure of LNPs was indistinguishable between the two methods, indicating that the fundamental mechanism of lipid self-assembly remains conserved regardless of synthesis platform (Figure 4G,H).^24,34^ These results confirm that particle quality, internal structure, and morphology are equivalent between HFM- and microfluidic-produced LNPs, with the primary distinctions being the degree of control over particle size and the number of particles generated within comparable time frames. Critically, the HFM system demonstrated superior scalability, with the production rate increasing proportionally to the number of fibers, where increasing from 2 to 78 fibers yielded a directly additive boost in LNP output. This scale-up was linear with respect to fiber count, enabling straightforward parallelization without the complex channel synchronization or pressure balancing required in microfluidic multiplexing (Figure 4J,K).^12^

The biological performance of LNPs synthesized via the HFM platform was rigorously evaluated both in vitro and in vivo, demonstrating comparable or enhanced functional outcomes relative to microfluidic-produced counterparts. In HEK293 cells transfected by EGFP mRNA, both HFM- and microfluidic-prepared LNPs produced comparable percentages of GFP-positive cells at 48 h, with similar GFP expression levels as measured by mean fluorescence intensity (MFI) (Figure 5A–C). Notably, the HFM method was also successfully applied to produce various LNP compositions; under identical processing conditions, formulations incorporating AA3-DLin, ALC-0315, and MC3 all demonstrated strong protein expression, with the AA3-DLin formulation achieving a transfection efficiency as high as 94% (Figure 5D–F). Current research results have confirmed that this process exhibits robust performance across various lipid systems, yet further optimization tailored to specific lipids may yield additional performance enhancements. Co-delivery of GFP plasmid DNA and mCherry mRNA in the same LNPs demonstrated dual expression efficiency greater than 80% for both constructs, confirming robust cargo delivery across multiple RNA species (Figure 5G,K). In vivo immunization studies using a COVID-19 mRNA vaccine encapsulated in HFM-produced AA3-DLin LNPs elicited potent humoral and cellular immune responses in mice, including robust spike-specific antibody titers and CD8^+^/CD4^+^ T cell activation, matching responses induced by microfluidic LNPs (Figure 6E–L). Collectively, these findings highlight the universality of the HFM approach, enabling efficient, reproducible nanoparticle production across multiple compositions, RNA types, and therapeutic applications.

From a biomanufacturing perspective, HFMs offer significant advantages in throughput, robustness, and process integration.^12,15,18^ As experimentally verified, by leveraging fiber parallelization techniques and linear throughput scalability, commercially available hollow fiber membrane modules can continuously produce LNPs at flow rates exceeding 1 L/min per unit, this value represents a theoretical scalability projection based on membrane parallelization rather than a directly demonstrated throughput, translating to roughly 10,000 doses per minute at a typical mRNA dose of 100 μg. This unprecedented production capacity could be pivotal in responding to emergent public health crises, where the ability to rapidly manufacture and deploy millions of doses can determine the speed and scale of outbreak containment. Furthermore, this throughput far surpasses that of a single CIJM or SHM, which typically processes around 12 mL/min, corresponding to approximately 120 doses per minute under similar conditions. In addition to high throughput, HFMs are compatible with in-line process analytical technologies (PAT), such as real-time dynamic light scattering (DLS), fluorescence quantification, and Raman spectroscopy, enabling tight process control. Features like antifouling coatings, automated cleaning protocols, and modular fiber replacement highlight the engineering feasibility for long-term continuous operation, although full validation under GMP conditions remains a subject of future investigation. Coupled with seamless integration of downstream purification methods, such as tangential flow filtration or inline diafiltration, the HFM platform presents a promising and scalable framework for end-to-end RNA lipid nanoparticle manufacturing.

While the HFM platform offers significant advantages, several challenges remain and require further study. Under prolonged operating conditions, the accumulation of lipids or proteins may lead to membrane fouling, thereby compromising the long-term stability of the system; this underscores the necessity of developing and implementing optimized antifouling surface treatment technologies or requisite cleaning methods. Although they are compatible with common lipid formulations, emerging lipids with distinct properties may demand tailored membrane materials and process adjustments. Current CFD and mass transfer models provide initial insights, but more comprehensive multiscale simulations are needed to fully understand the complex mixing and nucleation dynamics.^20,21,25^ Exploring advanced synthesis regimes, including hybrid membrane designs and dynamic flow conditions, could further enhance the control over nanoparticle formation. Additionally, real-world adoption will depend on integrating continuous downstream processing and robust PAT for real-time monitoring to ensure consistent GMP-scale production. Importantly, coupling multiple HFM modules in series with inline diafiltration and purification technologies holds great potential to enable fully continuous closed-loop manufacturing workflows for RNA therapeutics and other nanomedicines.

In conclusion, nanopore-mediated mixing via porous hollow fiber membranes represents a transformative advance in lipid nanoparticle manufacturing. By leveraging nanoscale jet injection through a massively parallel pore network, this platform achieves precise control over particle size, polydispersity, and RNA encapsulation while enabling continuous production at industrially relevant throughput. This technology has successfully realized the transition from microchannel-based mixing to nanoporous-membrane-based mixing, significantly shortening the characteristic mixing length scale and substantially increasing the interfacial area density. Building upon this foundation, a unique mixing mechanism has been identified, distinguished by rapid solvent exchange processes and mass transport occurring under confined conditions. Experimental and computational validation confirms the platform’s superiority over traditional microfluidic systems in scalability, consistency, and RNA payload integrity.^5,20,24,25,35^ The generalizability of this approach extends to a wide range of self-assembled nanostructures, positioning HFM reactors as a next-generation standard for scalable, high-performance nanomedicine manufacturing. As demand for mRNA vaccines and advanced RNA therapeutics continues to grow, the integration of HFM-based manufacturing offers a scalable, precision-engineered solution poised to support both preclinical investigation and commercialized global deployment, rapidly accelerating the transition from laboratory research to global clinical supply chains.

## Methods

### Materials

Hollow fiber membranes (HFMs) (10 kDa mPES­(C02-E010–10-N), 100 kDa mPES­(C02-E100–10-N), 750 kDa mPES­(C02-E750–10-N), 0.1 μm ME­(C02-M10U-06-N), and 100 kDa mPES with 12 fibers (T02-E100–10-N), 100 kDa mPES with 78 fibers­(S02-E100–10-N)) were all purchased from Repligen. (6Z,9Z,28Z,31Z)-Heptatriaconta-6,9,28,31-tetraen-19-yl4­(dimethylamino) butanoate (DLin-MC3-DMA) was purchased from Ambeed (IL, USA). ALC-0315 lipid was obtained from BroadPharm. PEGylated lipids 1,2-dimyristoyl-racglycero-3-methoxypolyethylene glycol-2000 (DMG-PEG) and phospholipids 1,2-dioleoyl-snglycero-3-phosphoethanolamine (DOPE) were obtained from Avanti Polar Lipids. Cholesterol, sodium acetate buffers, succinate buffer, citrate buffer, Cell Counting Kit-8 (CCK-8), anhydrous magnesium sulfate, and dialysis kits (MWCO 3.5 kDa) were all available from Sigma-Aldrich (St. Louis, MO, USA). All the cell culture reagents such as Dulbecco’s modified Eagle medium (DMEM), RPMI 1640 medium, fetal bovine serum (FBS), Opti-MEM reduced serum medium, 0.25% trypsin-EDTA (1×), penicillin-streptomycin, etc. were obtained from Gibco (Paisley, UK). Reporter gene-encoded mRNAs (mCherry and EGFP) and plasmid (GFP) were obtained from TriLink Biotechnologies. The spike mRNA was obtained from System Biosciences. All the other general chemicals and reagents were obtained from Sigma-Aldrich and VWR (Radnor, PA, USA).

### LNP Preparation Using Hollow Fiber Membranes (HFM)

LNPs were synthesized using a hollow fiber membrane (HFM) system. Organic and aqueous phases were prepared as described above and delivered using two Knauer Azura P4.2 pumps connected to Repligen MiniKros HFMs. Before each experiment, the HFM module was flushed with 100% ethanol to wet the pores, rinsed with nuclease-free water, and equilibrated with the respective feed solutions. During operation, the organic lipid solution in ethanol was introduced into the bore side, while the aqueous buffer containing mRNA was circulated on the shell side. The bore outlet was sealed, forcing the organic stream to permeate radially through the pores into the surrounding aqueous flow, where rapid solvent exchange induced nanoparticle formation at the liquid–liquid interface.

Continuous production was established by connecting feed reservoirs to the pumps and applying back-pressure at the shell outlet to maintain stable transmembrane flow. Process parameters were adjusted across total flow rates (TFRs) of 0.4–12 mL/min and aqueous-to-organic flow rate ratios (FRRs) of 1:1 to 5:1. The collected suspensions were incubated at 25 °C for 20 min to allow particle maturation, followed by purification through dialysis under the same conditions described for microfluidic-prepared LNPs.

### HFM-Based Continuous Synthesis, Purification, and Buffer Exchange of LNPs

LNP suspensions were produced as described above and then purified using hollow fiber membranes (HFMs) operated in series with a peristaltic pump and a solution reservoir. The shell compartment, pre-equilibrated with detergent solution, enabled countercurrent buffer exchange: sample flowed through the lumen, while permeate exited via the shell-side outlet. Complete buffer substitution was achieved over three iterative wash cycles with matched-volume buffer exchange, simultaneously facilitating ethanol removal through transmembrane equilibration.

### LNP Preparation Using Microfluidics

Lipid nanoparticles (LNPs) were also prepared using a microfluidic mixing approach. The organic phase consisted of AA3-DLin, DOPE, cholesterol, and DMG-PEG2000 at a molar ratio of 40:40:25:0.5, dissolved in ethanol. The aqueous phase contained luciferase-encoded mRNA (mLuc), mCherry-encoded mRNA (mCherry), or GFP plasmid DNA in 25 mM sodium acetate buffer (pH 5.0, adjusted with 0.1 M HCl/NaOH). Both phases were loaded into syringe pumps (NE-1000) and infused into a microfluidic chip (LabSmith, Herringbone MicroMixer Fluidic 187) at a fixed nitrogen-to-phosphate (N:P) ratio of 6:1. The total flow rate (TFR) was varied between 0.4 and 12 mL/min, with aqueous-to-organic flow rate ratios (FRRs) ranging from 1:1 to 5:1. The resulting LNP suspensions were incubated at 25 °C for 20 min and dialyzed against 1× phosphate-buffered saline (PBS, pH 7.4) using a 3.5 kDa molecular weight cutoff (MWCO) dialysis membrane (Pur-A-Lyzer Maxi Dialysis Kit) for 6 h with three buffer exchanges. LNPs were stored at 4 °C for up to 72 h prior to characterization.

### HFM Characterization

The internal morphology of the HFMs was examined by scanning electron microscopy (SEM). Fibers were cryogenically fractured by immersion in liquid nitrogen for 20 s, sputter-coated with Au/Pd using an EMS 150 TES coater, and imaged with a JEOL JSM-7900F field-emission SEM instrument at 2 kV accelerating voltage. Cross-sectional and surface micrographs were acquired at magnifications ranging from 100× to 20,000×.

### Computational Fluid Dynamics (CFD) Simulations

Computational simulations were performed using COMSOL Multiphysics to investigate the early-stage hydrodynamic interaction between the injected organic phase and the surrounding aqueous flow in the hollow fiber membrane (HFM) system. A two-dimensional (2D) pore-channel model was constructed to approximate the nanoporous membrane architecture as an array of uniformly distributed injection sites with pore diameters ranging from 5 to 200 nm.

Governing Equations: The flow field was described using the incompressible laminar Navier–Stokes equations

$$\begin{array}{l} \rho \left(\frac{\partial u}{\partial t}+u\cdot \nabla u\right)=-\nabla p+\nabla \cdot \tau +F\\ \rho \nabla \cdot u=0 \end{array}$$

where

ρ is the fluid density,

**u** is the velocity vector,

*p* is the pressure,

**F** represents external body forces:

Gravitational and other body forces were neglected in the present study.

The viscous stress tensor **τ** was defined using the Newtonian constitutive relation:

$$\tau =\mu \left(\nabla u+{\left(\nabla u\right)}^{T}\right)$$

where μ is the dynamic viscosity.

Given the nanoscale characteristic dimensions (5–200 nm) and low flow velocities, the Reynolds number is much smaller than unity (*Re* ≪ 1), indicating a viscous-dominated laminar flow regime. Therefore, no turbulence model was applied.

Level Set Formulation: The transient interaction between the injected organic phase and the aqueous phase was described using a Level Set formulation. The Level Set variable ϕ­(implemented as *phils* in COMSOL) evolves according to

$$\frac{\partial \phi }{\partial t}+u\cdot \nabla \phi =\gamma \nabla \cdot \left(\epsilon_{\mathrm{ls}}\nabla \phi -\phi \left(1-\phi \right)\frac{\nabla \phi }{\left|\nabla \phi \right|}\right)$$

where:

ϕ is the level set function (0 ≤ ϕ ≤ 1), representing the spatial distribution of the two fluid regions,

γ is the reinitialization parameter controlling numerical stabilization,

ϵ_ls_ is the interface thickness parameter,

**u** is the velocity vector.

The right-hand side consists of a diffusion-like term that controls the interface thickness and a compression term that maintains interface sharpness during numerical advection.

It should be noted that the level set variable is not interpreted as an ethanol concentration or mass fraction field. Rather, it serves as a numerical marker used to visualize the transient spatial distribution of the injected organic-rich region and to compare hydrodynamic interaction patterns under different pore size conditions.

The interaction area was calculated through surface integration over this transition region

$$A_{\mathrm{int}}=\int_{\Omega }I\left(0.4<\phi <0.6\right)d\Omega$$

where *A*_int_ is the interaction area, Ω denotes the computational domain, and I() is an indicator function that equals 1 when the condition 0.4 < ϕ < 0.6 is satisfied and 0 otherwise.

To enable comparison across different conditions, the interaction area was further normalized by the total computational domain area

$$A_{\mathrm{rel}}=\frac{A_{\mathrm{int}}}{A_{\mathrm{domain}}}$$

where *A*_rel_ represents the dimensionless relative contact surface and *A*_domain_ is the total computational domain area.

Boundary and initial conditions: The aqueous phase was introduced through the main channel inlet, representing the bulk flow, while the organic phase was injected through nanopore openings to mimic transmembrane transport. Velocity boundary conditions were prescribed at all inlets, a constant-pressure condition was imposed at the outlet, and no-slip boundary conditions were applied to all solid walls. The system was assumed to be incompressible and isothermal at 293.15 K.

Numerical implementation: Transient simulations were performed using a time-dependent solver with a time step of 0.0001s. The computational domain was discretized using a physics-controlled mesh consisting of approximately 17,000–20,000 triangular elements. Mesh independence was verified by comparing velocity fields and Level Set distributions across multiple mesh densities, ensuring that further mesh refinement did not significantly affect the simulation results.

### Design of Experiments (DoE) Analysis

A three-factor Box–Behnken design was implemented in JMP Pro 16 (SAS Institute) to examine the effects of membrane pore size (5–200 nm), flow rate ratio (FRR: 1:1–5:1), and total flow rate (TFR: 0.4–12 mL/min) on LNP size and polydispersity index (PDI). The design included 15 experimental runs for second-order interaction modeling. Regression analysis (least-squares method) was used to quantify factor effects, with significance set at *p* < 0.05. Response surface models were generated to visualize parameter interactions and identify optimal formulation conditions.

### LNP Characterization

Hydrodynamic size, polydispersity index (PDI), and zeta potential were measured using dynamic light scattering (DLS) and Zetasizer Nano ZS (Malvern Panalytical, UK) at 25 °C. All measurements were performed in triplicate, and results are reported as mean ± standard deviation (SD).

mRNA encapsulation efficiency (EE) was quantified with the Quant-iT RiboGreen RNA Assay Kit (Invitrogen). LNP samples (10 μL) were diluted in TE buffer and incubated with RiboGreen reagent (1:200 dilution, 5 min, dark). Fluorescence was measured at 480 ex/520 em nm using a Tecan Infinite M200 plate reader to quantify unencapsulated (free) mRNA. Total mRNA content was determined by disrupting LNPs with 2% Triton X-100 (10 min, 25 °C). EE (%) was calculated as

$$mRNA\;(encapsulation\;efficiency\;\%)=\frac{\left(\mathrm{total}\;mRNA\,-free\;mRNA\right)}{total\;mRNA}\times 100\%$$

LNPs prepared by microfluidic and HFM methods were lysed by using 5% Triton X-100 for 5 min to release encapsulated mRNA. Following lysis, samples were diluted with an equal volume of PBS and purified by using ultrafiltration (three washing cycles) to remove residual lipids and surfactants. The recovered mRNA was then analyzed for integrity using capillary electrophoresis (CE, Qsep100, Bioptic).

For stability tests, LNPs prepared using the HFM platform were evaluated for storage stability under clinically relevant conditions. For refrigerated storage, LNP samples were stored at 4 °C and characterized at predetermined time points (Day 0, 7, and 14). For frozen storage, LNPs were stored at −20 °C in the presence of 10% (w/v) sucrose as a cryoprotectant. To assess freeze–thaw stability, LNP samples were subjected to multiple freeze–thaw cycles (−20 °C to room temperature), with each cycle consisting of complete freezing followed by thawing to ambient temperature. After each cycle, samples were analyzed for physicochemical properties. At each condition and time point, particle size and PDI were measured using DLS, and EE was determined using the RiboGreen assay.

### Transmission Electron Microscopy (TEM)

For negative-stain TEM, 5 μL of LNP suspension was deposited onto 400-mesh carbon-coated copper grids (Ted Pella), blotted after 1 min, and stained with 2% uranyl acetate (Electron Microscopy Sciences) for 30 s. This staining–blotting cycle was repeated three times before air-drying. Imaging was performed using a JEOL JEM-F200 field emission TEM operated at 200 kV. For cryo-TEM, 5 μL aliquots were applied onto glow-discharged 300-mesh lacey carbon grids (Ted Pella) and vitrified in liquid ethane. Grids were stored in liquid nitrogen and imaged at <90 K using a JEOL 2100Plus TEM with a cryogenic tomography holder.

### Small-Angle X-ray Scattering (SAXS)

SAXS measurements were performed at Brookhaven National Laboratory (NY, USA) using a SAXSLabs system equipped with a Cu Kα source (λ = 1.5418 Å) and a Dectris Pilatus 300 K detector. Freshly prepared LNP samples were loaded into 1.0 mm borosilicate glass capillaries and measured at 25 °C. Two-dimensional scattering patterns were azimuthally averaged and converted into one-dimensional scattering profiles after buffer subtraction using SAXSGUI and in-house scripts. Instrument calibration was performed using silver behenate.

### Cells and Animals

HEK293 cells (ATCC) were cultured in Dulbecco’s modified Eagle medium (DMEM; Gibco) supplemented with 10% fetal bovine serum (FBS; Gibco) and 1% penicillin–streptomycin (Gibco) at 37 °C in a humidified atmosphere with 5% CO_2_. For in vitro transfection, cells were seeded in 24-well plates at a density of 1.0 × 10^5^ cells per well and allowed to adhere overnight in 800 μL complete growth medium. LNP formulations were prepared freshly before use and diluted in sterile PBS. For each well, 50 μL of LNP suspension containing 1–2 μg of encapsulated mRNA or plasmid DNA was gently added dropwise to the medium (final volume 850 μL per well).

BALB/c mice (6–8 weeks old) were obtained from The Jackson Laboratory. The mice were housed in a specific pathogen-free animal facility under controlled environmental conditions, with the temperature maintained at 20–25 °C, relative humidity maintained at 40–70%, and a 12 h light/dark cycle. The mice had ad libitum access to standard chow and water. All animal procedures were conducted in accordance with the ethical standards and guidelines approved by the Institutional Animal Care and Use Committee of Rutgers University (eIACUC Approval No. 999901065).

### Biodistribution Study

mLuc-loaded LNPs prepared using microfluidic and HFM methods were intravenously administered to BALB/c mice. At 6 h post-injection, mice were anesthetized and intraperitoneally injected with 200 μL of D-luciferin solution (15 mg/mL). Bioluminescence signals were then quantified and analyzed using an IVIS Spectrum imaging system (PerkinElmer).

### Mouse Vaccination Study

Mice (*n* = 5 per group) were vaccinated intramuscularly in the left hind leg with 2 or 10 μg of spike mRNA–loaded AA3-DLin LNPs (5 wt % sucrose). Placebo controls received 100 μL of empty LNPs. Blood was collected via orbital sinus puncture, and spleens were harvested on day 30 for immune response analyses. Serum was separated by centrifugation at 3000*g* and heat-inactivated at 56 °C for 30 min.

### SARS-CoV-2 ELISA

Spike-specific antibody titers were measured using ELISA. Briefly, 96-well MaxiSorp plates were coated overnight at 4 °C with 0.1 μg of recombinant SARS-CoV-2 spike protein (Sino Biological). Plates were washed three times with 200 μL wash buffer (Thermo Fisher Scientific), blocked with 200 μL assay buffer for 1 h at room temperature, then incubated with 100 μL of serially diluted serum samples for 2 h. After triplicate washes, wells were incubated with HRP-conjugated goat anti-mouse IgG (1:3000) for 1 h, followed by 50 μL of TMB substrate and 50 μL stop solution. Absorbance at 450 nm was measured using a Tecan plate reader. End point titers were defined as the highest reciprocal serum dilution with an absorbance >2.1-fold above control.

### Intracellular Cytokine Staining (ICS)

Splenocytes (1 × 10^6^ cells/well) were stimulated with SARS-CoV-2 spike peptide pools (2 μg/mL, 6 h) in the presence of BD GolgiPlug at 37 °C. Cells were washed three times, stained with LIVE/DEAD Cell Stain Kit (Invitrogen), and labeled with anti-CD3, anti-CD4, and anti-CD8a antibodies (1:100 each). Following fixation and permeabilization with BD Cytofix/Cytoperm, intracellular cytokine staining was performed with antibodies against IFN-γ, IL-2, TNF, and IL-4 (1:100 each). Flow cytometry was performed on a BD LSRII instrument, with 100,000 events collected and analyzed using FlowJo software.

## Supplementary Material



## Data Availability

All data are available in the main text or the Supporting Information.

## Notes

Formulation optimization, process-parameter analysis, HFM pore-density analysis, continuous purification and ethanol-removal characterization, AA3-DLin synthesis, FACS gating strategies, in vitro transfection analysis, mRNA integrity analysis, LNP stability, biodistribution, long-term continuous operation, cryo-TEM morphology, and mass-fraction-based simulation data (Figures S1–S24) and buffer-condition data sets and mixing-platform comparison (Tables S1–S4) (PDF)
