# Supplementary material for: Nanopore-Mediated Assembly Enables Precise and Continuous Synthesis of mRNA-Encapsulated Lipid Nanoparticles for COVID-19 Vaccines
Source: ACS Nano. 2026 Jul 16;20(29):20513–29. doi: 10.1021/acsnano.6c00729 (PMC13421963; doi:10.1021/acsnano.6c00729)
Supplement: Supplementary file 1 [file nn6c00729_si_001.pdf]

Supporting Information

Nanopore-Mediated Assembly Enables Precise and Continuous Synthesis of mRNA-Encapsulated Lipid Nanoparticles for COVID-19 Vaccines

Zhixiang Liu[1], William Stewart[1], Yilong Teng[2], Yufeng Song[1], Maoping Tang[2], Yuxuan Guo[2], Xue-Qing Zhang*[2], Kamalesh K. Sirkar*[1], Xiaoyang Xu*[1,3]

Affiliations:

[1]Department of Chemical and Materials Engineering, New Jersey Institute of Technology; Newark, 07103, USA.

[2]Shanghai Frontiers Science Center of Drug Target Identification and Delivery, School of Pharmaceutical Sciences, National Key Laboratory of Innovative Immunotherapy, Shanghai Jiao Tong University; Shanghai, 200240, P. R. China.

[3]Department of Biomedical Engineering, New Jersey Institute of Technology; Newark, 07103, USA.

*Corresponding authors.

Fig. S1.

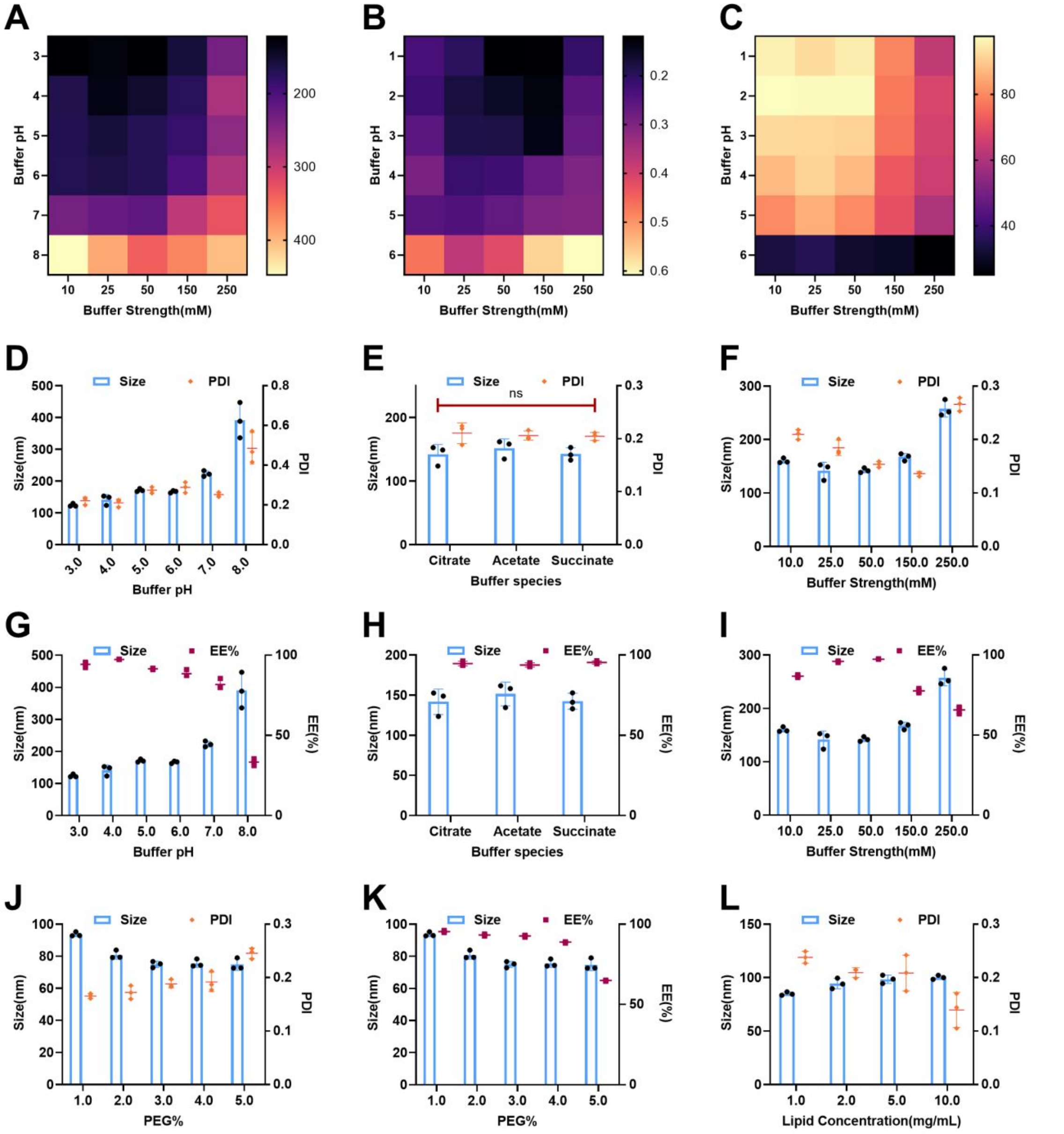

Influence of chemical composition factors on LNP characteristics via HFM production. (**A-C**) Heat map detailing influence of buffer pH and molarity on (**A**) Particle size, (**B**) PDI, and (**C**) Encapsulation efficiency. (**D-F**) Effect of (**D**) buffer pH, (**E**) buffer species, and (**F**) buffer molarity on particle size and PDI of LNPs produced via HFM method. (**G-I**) Effect of (**G**) buffer pH, (**H**) buffer species, and (**I**) buffer molarity on particle size and encapsulation efficiency of LNPs produced via HFM method. (**J-K**) Effect of Lipid-PEG molar percentage on particle size and (**J**) PDI or (**K**) encapsulation efficiency of LNP's produced via HFM method. (**L**) Effect of total lipid concentration on particle size and PDI of LNP's produced via HFM method.

Fig. S2.

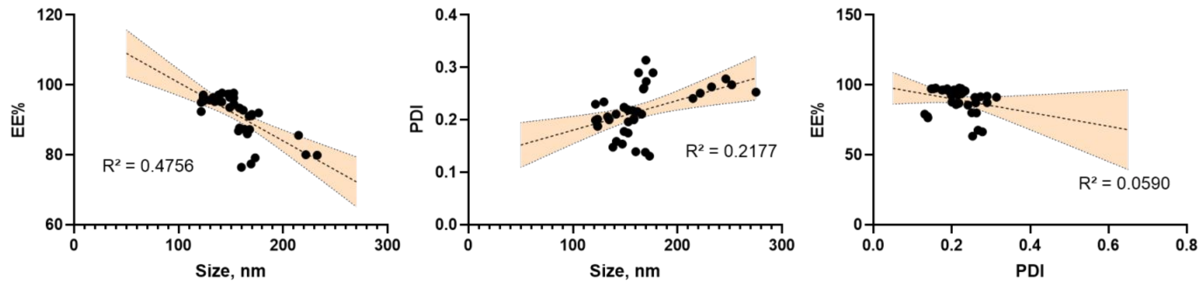

Relationship analysis of particle size, PDI, and EE%.

The analysis shows the correlation between particle size, PDI and EE%. From the R^2 values, there is no obvious correlation between the three parameters.

Fig. S3.

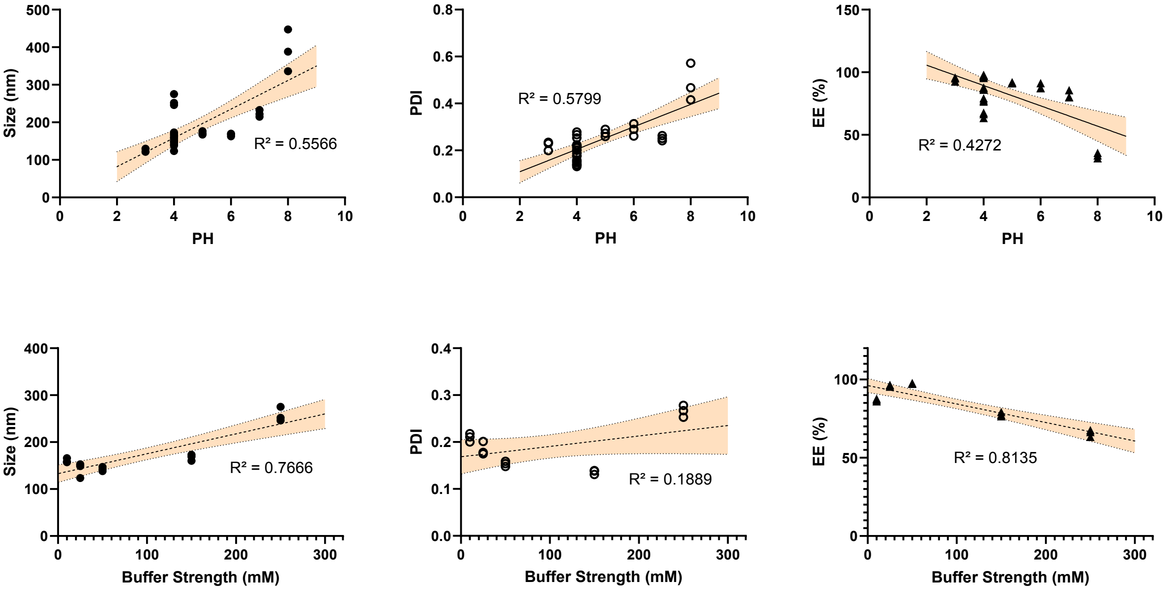

Relationship analysis between buffer pH and buffer strength with particle size, PDI, and EE%.

Relationship analysis of buffer pH and ionic strength with LNP properties revealed clear trends. Increasing buffer pH led to larger particle size ($R^2 = 0.5566$) and higher PDI ($R^2 = 0.3759$), accompanied by reduced encapsulation efficiency (EE%, $R^2 = 0.4627$). Similarly, increasing buffer strength enlarged particle size ($R^2 = 0.7666$) with only minor effects on PDI ($R^2 = 0.1369$) but decreased EE% ($R^2 = 0.6215$). Together, these results indicate that alkaline and high-salt conditions favor particle growth while compromising encapsulation efficiency.

Fig. S4.

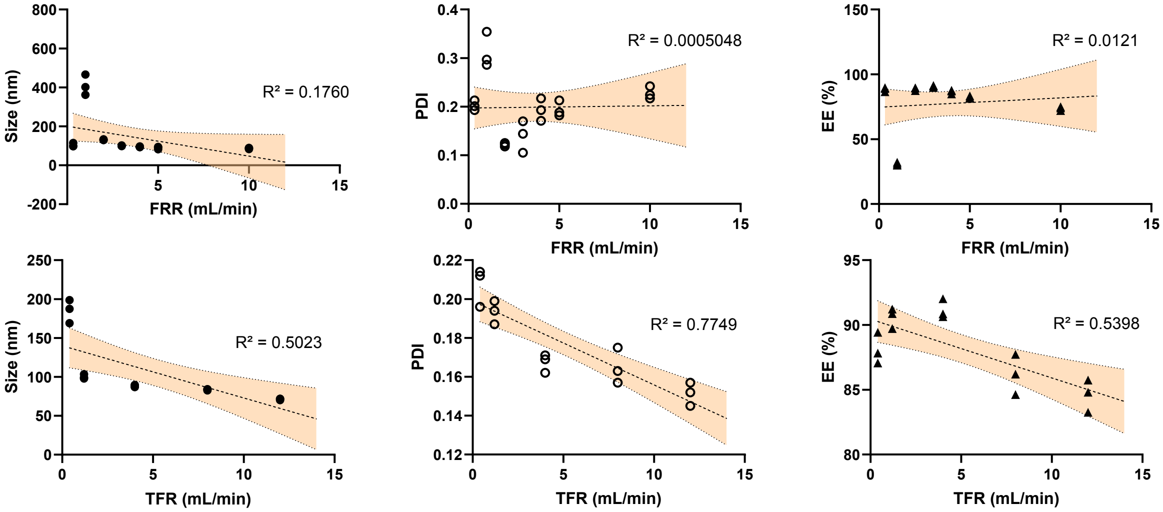

Relationship analysis between TFR and FRR with particle size, PDI, EE%.

Increasing TFR led to a modest reduction in particle size and EE%, with little impact on PDI. In contrast, higher FRR produced a pronounced decrease in both particle size and EE% while leaving PDI largely unaffected. These findings suggest that greater mixing intensity, especially at elevated FRR, promotes the formation of smaller particles but compromises encapsulation efficiency.

Fig. S5.

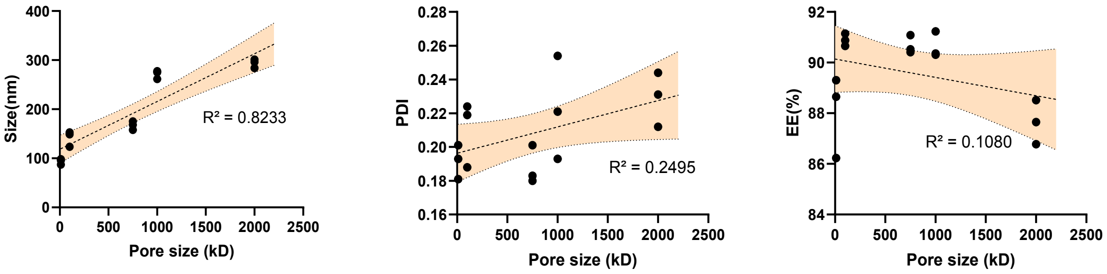

Relationship analysis between HFM pore size and particle size, PDI and EE%.

Increasing pore size strongly correlated with larger particle size ($R^2 = 0.8233$) and modestly increased PDI ($R^2 = 0.2266$), while having no significant effect on encapsulation efficiency (EE%, $R^2 = 0.1698$). These findings indicate that larger-pore membranes promote the formation of bigger, less uniform particles, yet preserve high encapsulation efficiency..

Fig. S6.

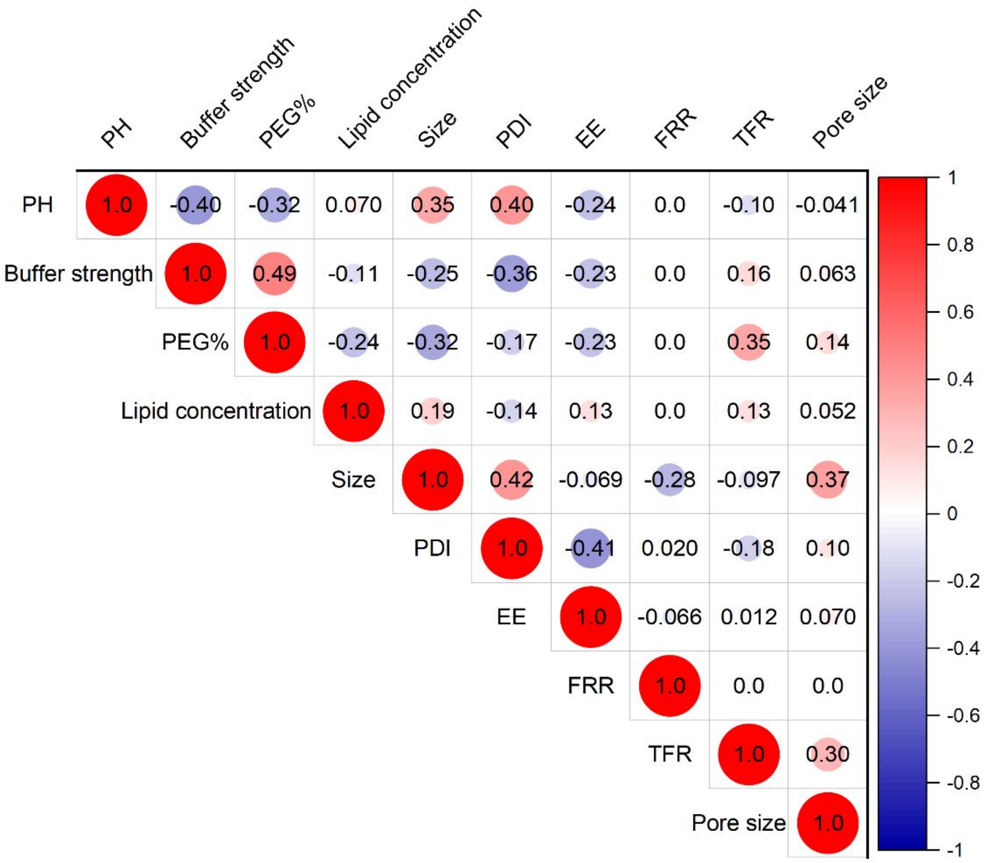

Correlation analysis of all influence parameters.

The correlation matrix revealed that particle size was positively associated with PDI (r = 0.42) and pore size (r = 0.37), but negatively correlated with FRR (r = –0.28). Encapsulation efficiency (EE%) showed a negative correlation with PDI (r = –0.41). Together, these results indicate that smaller pore sizes and lower FRR promote the formation of smaller, more uniform particles, and that reduced PDI is linked to improved encapsulation efficiency.

Fig. S7.

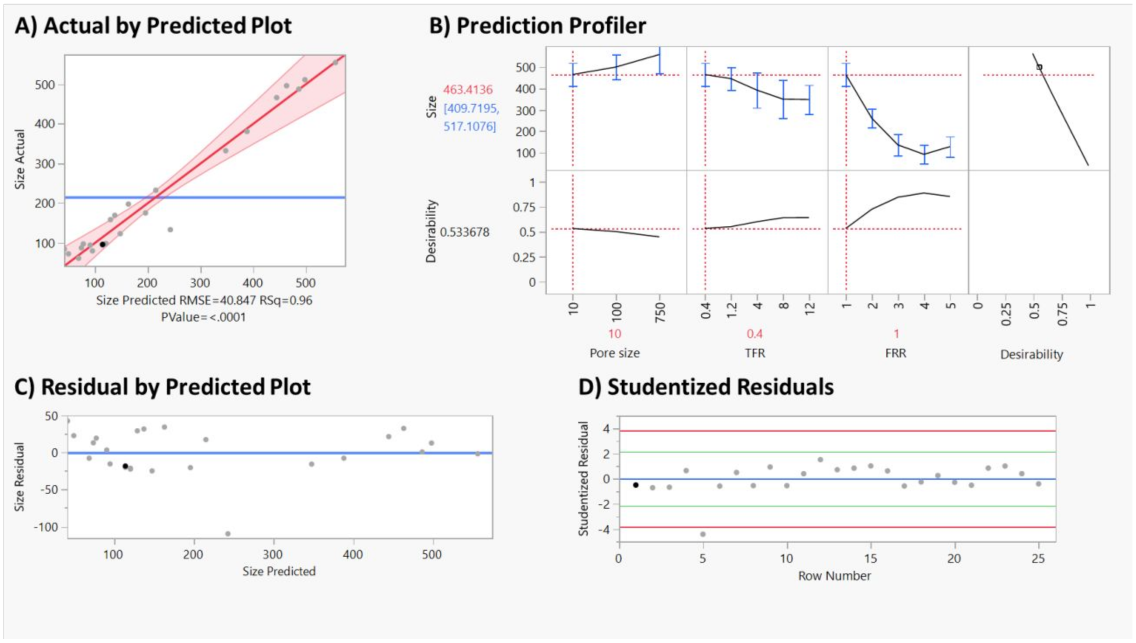

Least squares response analysis for particle size change in response to FRR, TFR and HFM Pore size. (A) Actual versus predicted particle size demonstrating strong agreement between experimental data and model predictions ($R^2 = 0.96$). (B) Prediction profiler showing the modeled effects of pore size, total flow rate (TFR), and flow rate ratio (FRR) on particle size, alongside the overall desirability score. (C) Residuals versus predicted size confirming the absence of systematic bias. (D) Studentized residuals indicating no significant outliers.

Fig. S8.

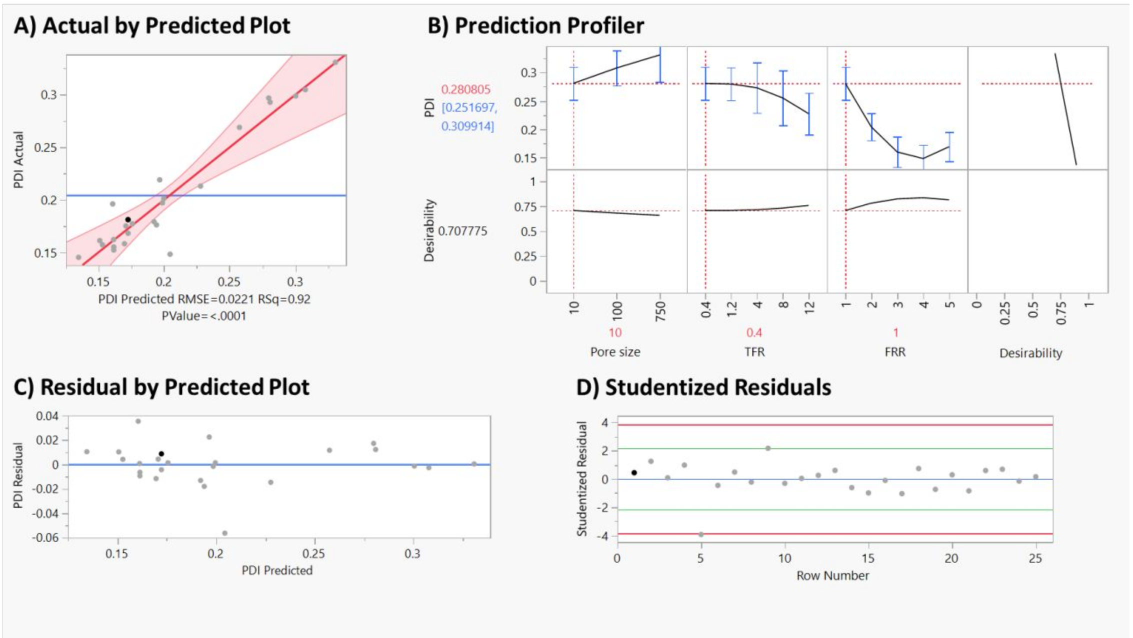

Least squares response analysis for PDI change in response to FRR, TFR and HFM Pore size. (A) Actual versus predicted polydispersity index (PDI) demonstrating strong agreement between model predictions and experimental data ($R^2 = 0.92$). (B) Prediction profiler showing the modeled effects of pore size, total flow rate (TFR), and flow rate ratio (FRR) on PDI, along with the overall desirability response. (C) Residuals versus predicted PDI confirming no systematic deviation. (D) Studentized residuals indicating the absence of significant outliers.

Fig. S9.

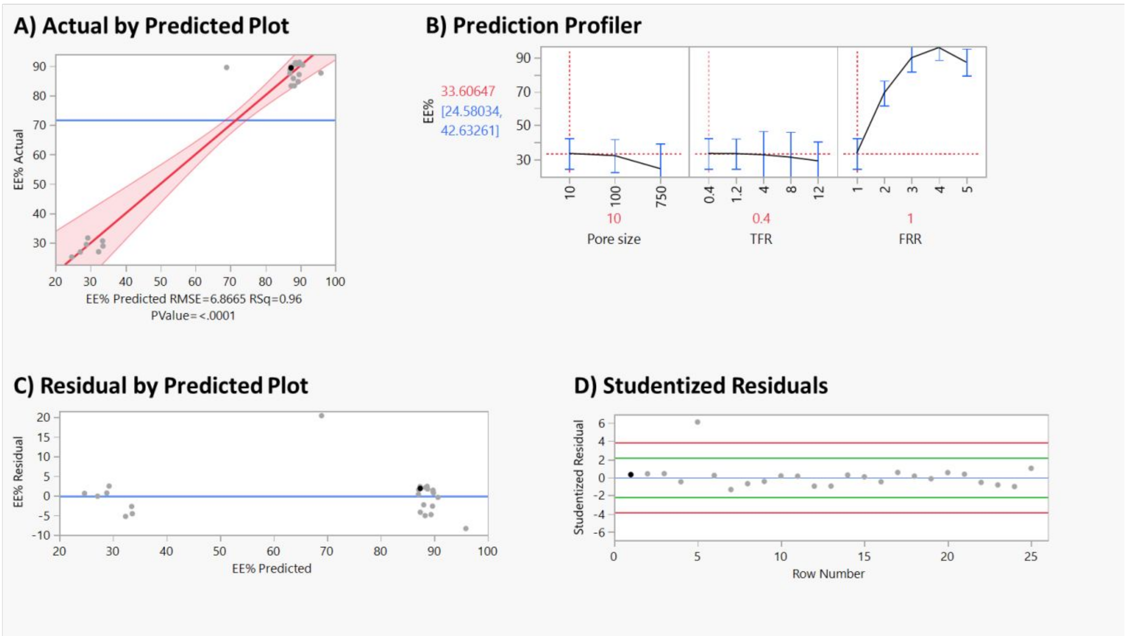

Least Squares response analysis for EE% change in response to FRR, TFR and Pore size. (**A**) Actual versus predicted encapsulation efficiency (EE%) showing strong agreement between model predictions and experimental data (R^2 = 0.96). (**B**) Prediction profiler illustrating the modeled effects of pore size, total flow rate (TFR), and flow rate ratio (FRR) on EE%. (**C**) Residuals versus predicted EE% confirming no systematic deviation. (**D**) Studentized residuals indicating the absence of significant outliers.

Fig. S10.

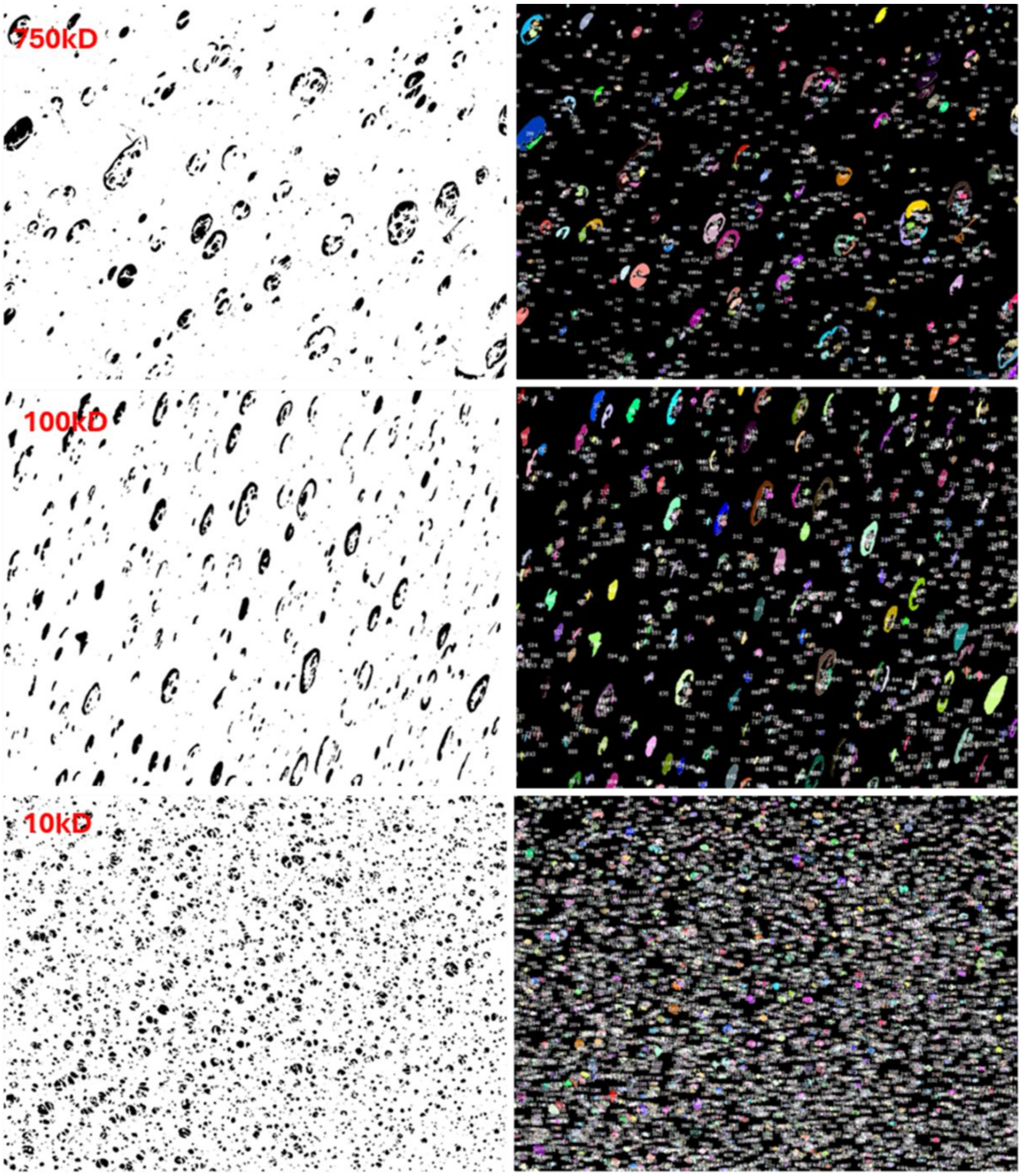

HFM	Count	Total Area, μm²	Pore density (per 100 μm²)
750kD HFM	895	8657.99	10.34
100kD HFM	909	1448.71	62.74
10kD HFM	4230	3202.77	132.07

HFM pore density analysis and calculation via ImageJ. ImageJ analysis of SEM micrographs showed that pore density increased as MWCO decreased: 10.3 pores/100 μm² for the 750 kDa HFM, 62.7 pores/100 μm² for the 100 kDa HFM, and 132.1 pores/100 μm² for the 10 kDa HFM

Fig. S11.

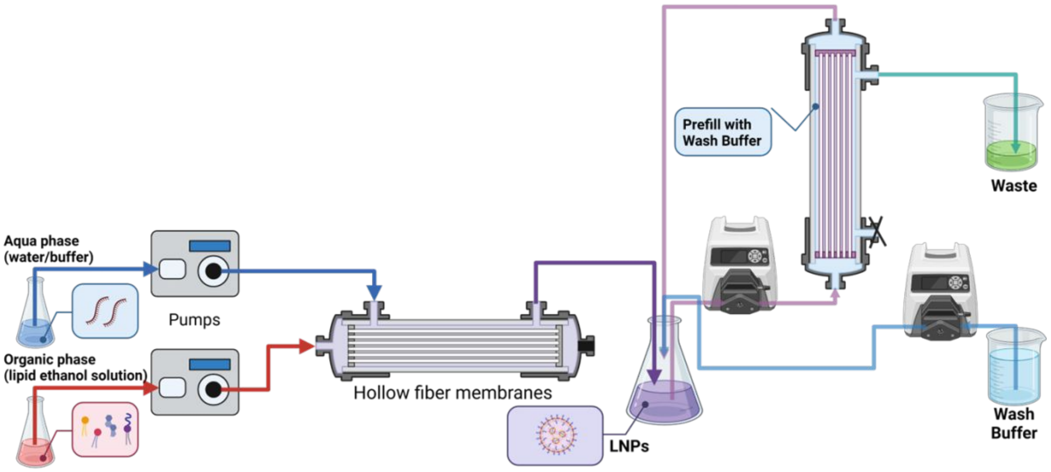

Schematic diagram for continuous LNP preparation, purification, and buffer exchange via HFM. To enable continuous LNP production, we implemented a two-stage tandem HFM process. In the first module, LNPs were synthesized and pooled in a collection reservoir. The suspension was then passed inline through a second module for purification and buffer exchange via eight diavolume cycles, achieving fully continuous production and downstream processing of LNPs.

Fig. S12.

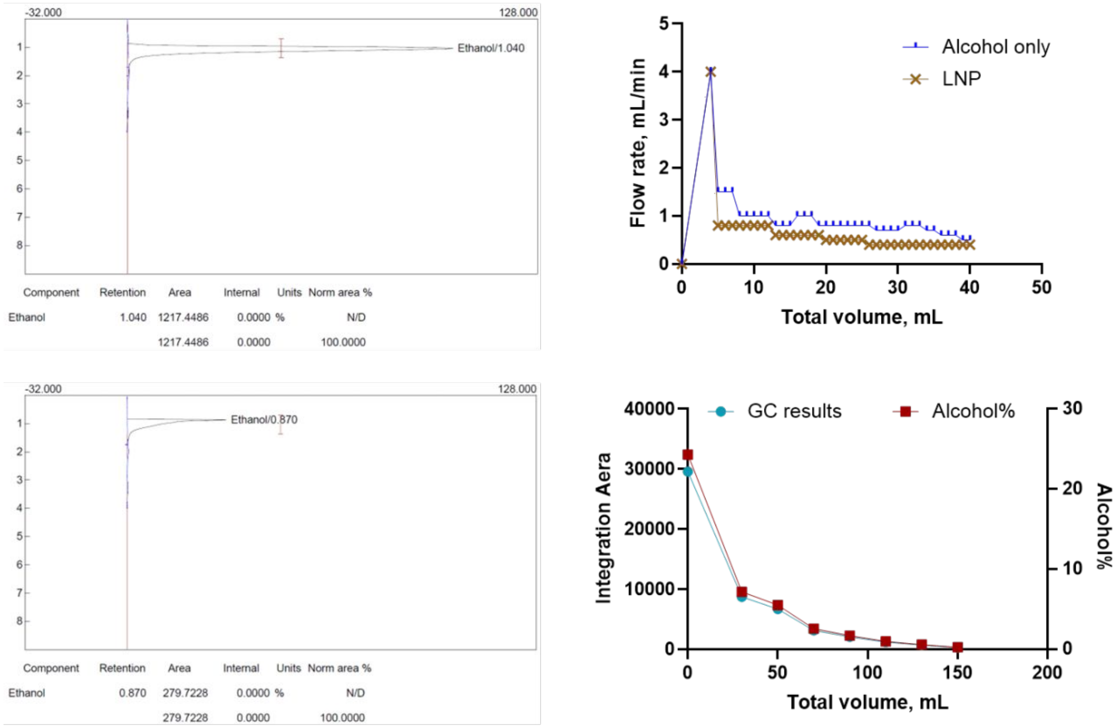

Ethanol removal and LNP purification during continuous LNP production.

Gas chromatography shows ethanol concentration decreased to <2% after 8× buffer exchange (diavolumes) and approached 0% after 12× diavolumes, confirming efficient solvent removal during LNP purification.

Fig. S13.

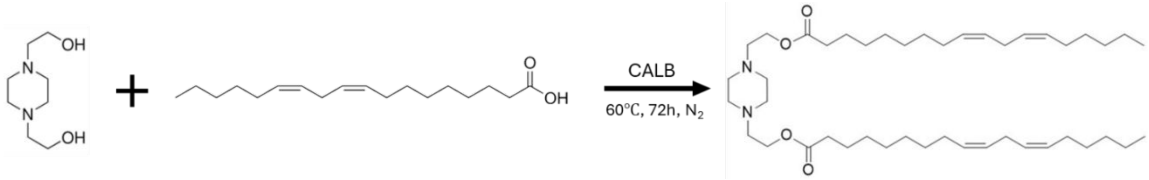

Synthesis of Ionizable Cationic Lipid AA3-Dlin.

The synthesis of ionizable cationic lipids was achieved via Candida antarctica lipase B (CALB)-catalyzed esterification between hydroxyl groups (−OH) of amino alcohols and carboxylic acid moieties (−COOH) of lipid acids. The procedure was conducted as follows: In a dual-neck round-bottom flask, 0.5 g of amino alcohol was precisely weighed and dissolved in 5 mL of anhydrous tetrahydrofuran (THF). A twofold molar excess of lipid acid relative to the amino alcohol was introduced, followed by the addition of 0.5 g of immobilized CALB as the biocatalyst. The reaction mixture was maintained at 60 °C under nitrogen atmosphere for 72 h to ensure complete esterification.

Post-reaction, the immobilized CALB was separated via centrifugation, and the supernatant was subjected to sequential purification steps. Residual lipid acid was neutralized by washing the crude product with saturated sodium bicarbonate ($NaHCO_3$) solution. The organic phase was then extracted with excess ethyl acetate, dried over anhydrous magnesium sulfate ($MgSO_4$), and filtered to remove desiccant particles. The resulting ethyl acetate solution containing the lipid product was concentrated using a rotary evaporator under reduced pressure. Finally, the purified lipids were vacuum-dried at ambient temperature for 48 h prior to characterization or application.

Fig. S14.

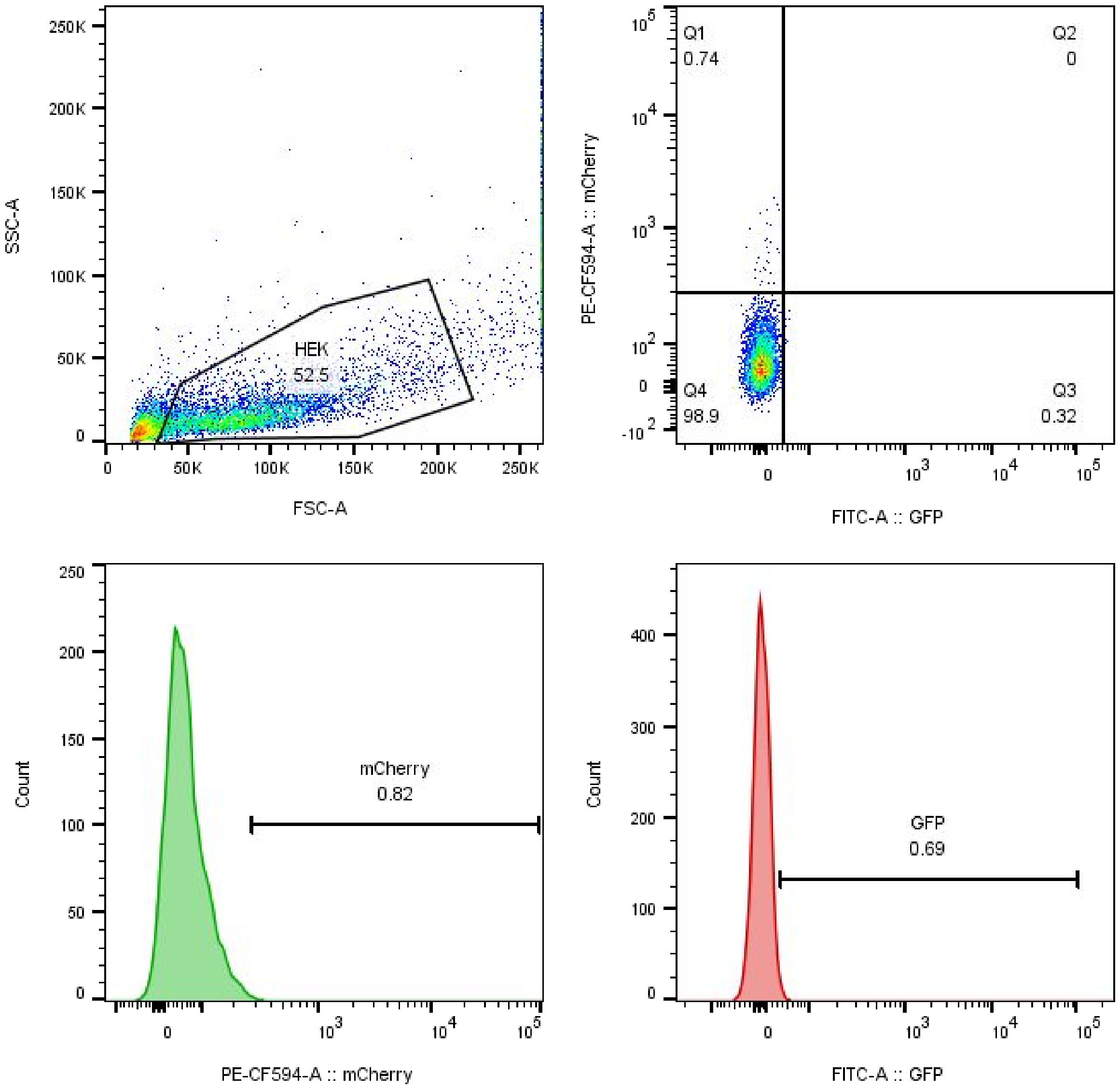

Negative control FACS gating strategy and quantitative analysis for in vitro GFP and mCherry transfection.

Fig. S15.

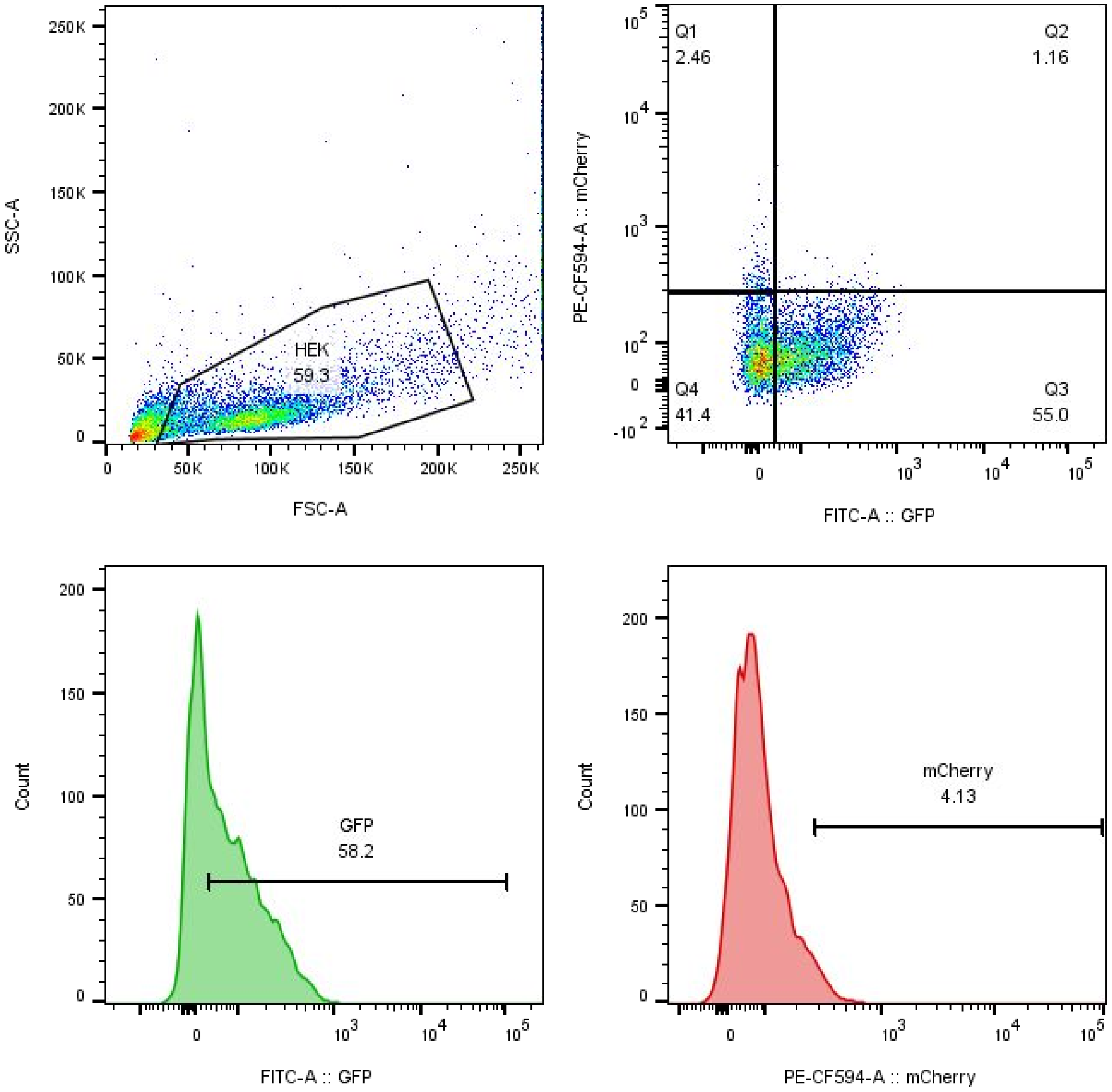

GFP positive FACS gating strategy and quantitative analysis for in vitro GFP and mCherry transfection.

Fig. S16.

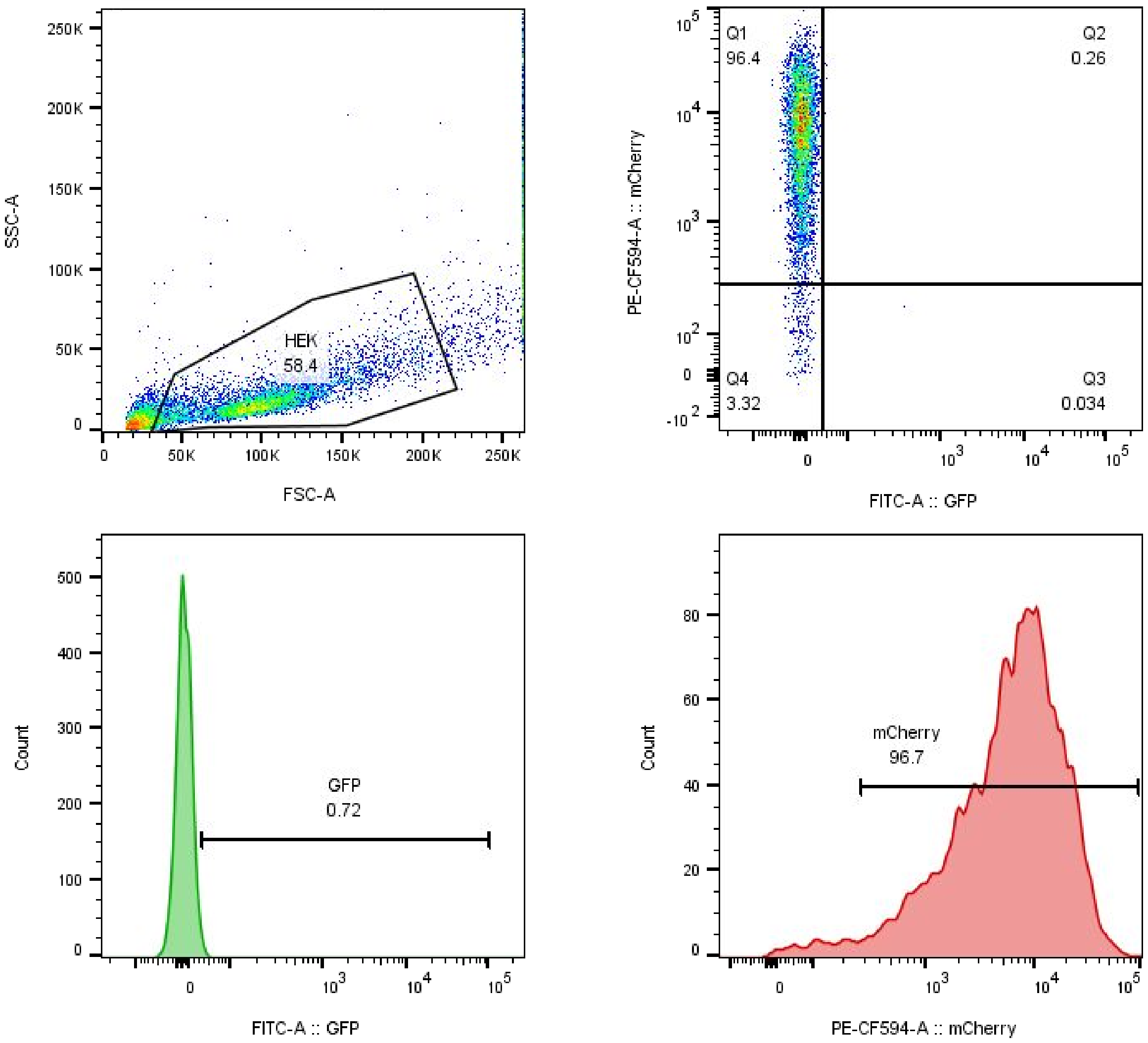

mCherry positive FACS gating strategy and quantitative analysis for in vitro GFP and mCherry transfection.

Fig. S17.

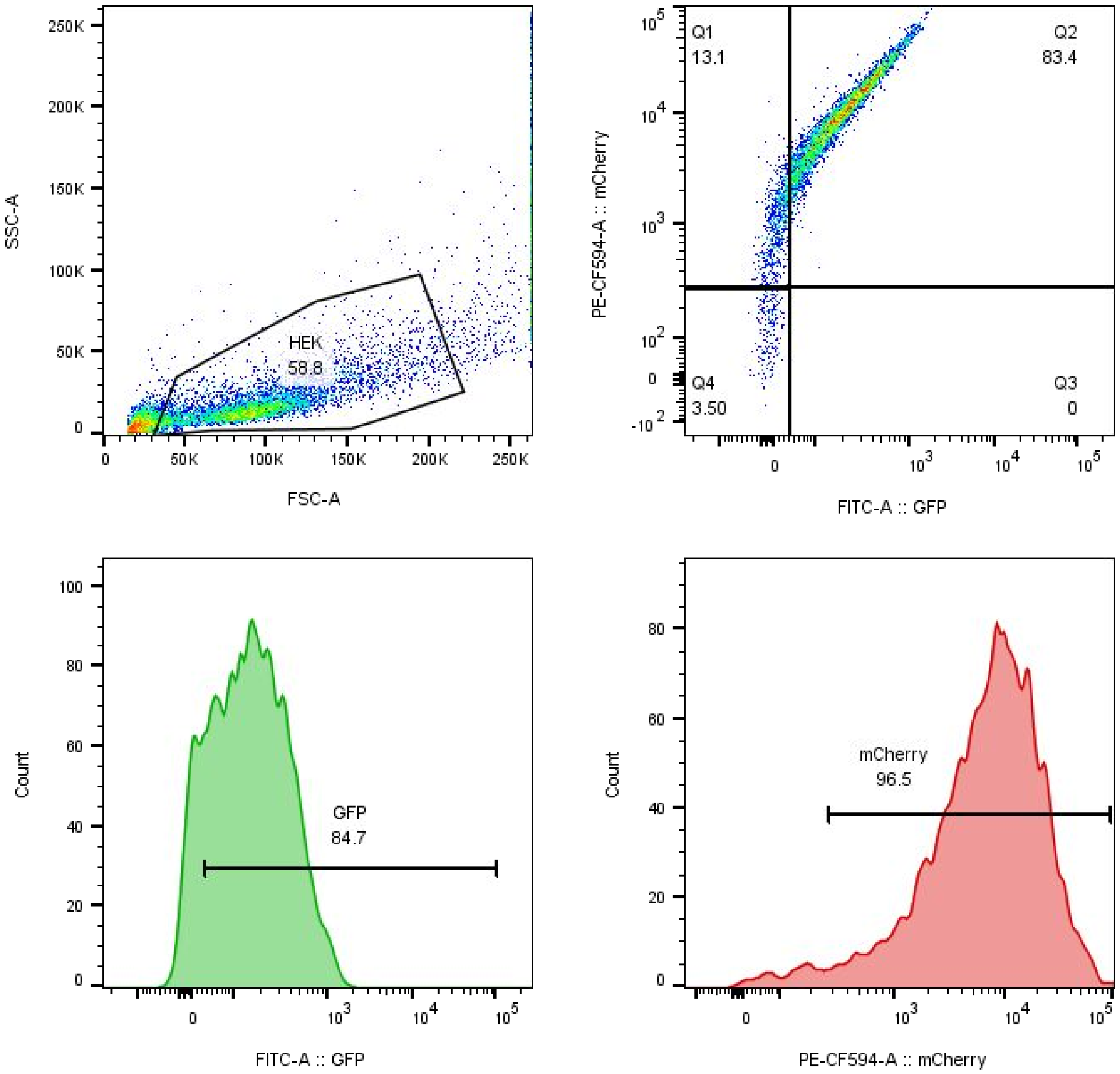

Codelivery of GFP and mCherry positive FACS gating strategy and quantitative analysis for in vitro GFP and mCherry transfection.

Fig. S18.

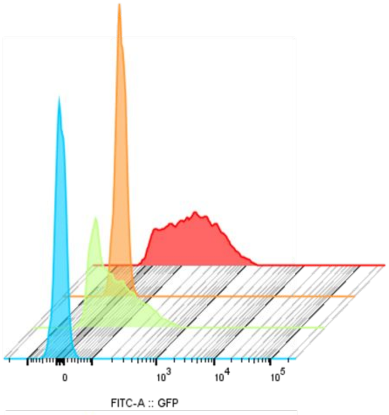

	Sample Name	Subset Name	Count	Mean : FITC-A	Geometric Mean : FITC-A
	Specimen_001_Control.fcs	*HEK*	*5251*	*1.89*	*1.88*
	Specimen_001_GFP.fcs	HEK	5928	93.3	69.0
	Specimen_001_mCherry.fcs	HEK	5837	2.28	2.23
	Specimen_001_GFP mCherry.fcs	HEK	5878	214	147

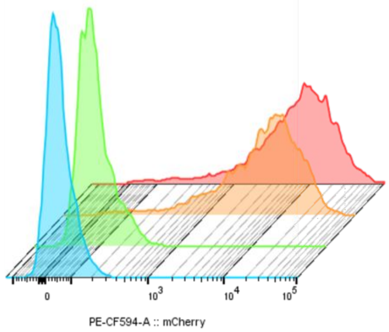

	Sample Name	Subset Name	Count	Mean : PE-CF594-A	Geometric Mean : PE-CF594-A
	Specimen_001_Control.fcs	*HEK*	*5251*	*66.9*	*61.1*
	Specimen_001_GFP.fcs	HEK	5928	97.4	86.2
	Specimen_001_mCherry.fcs	HEK	5837	9136	5105
	Specimen_001_GFP mCherry.fcs	HEK	5878	10788	5892

FACS histogram overlay and quantitative analysis for in vitro GFP and mCherry transfection.

Fig. S19.

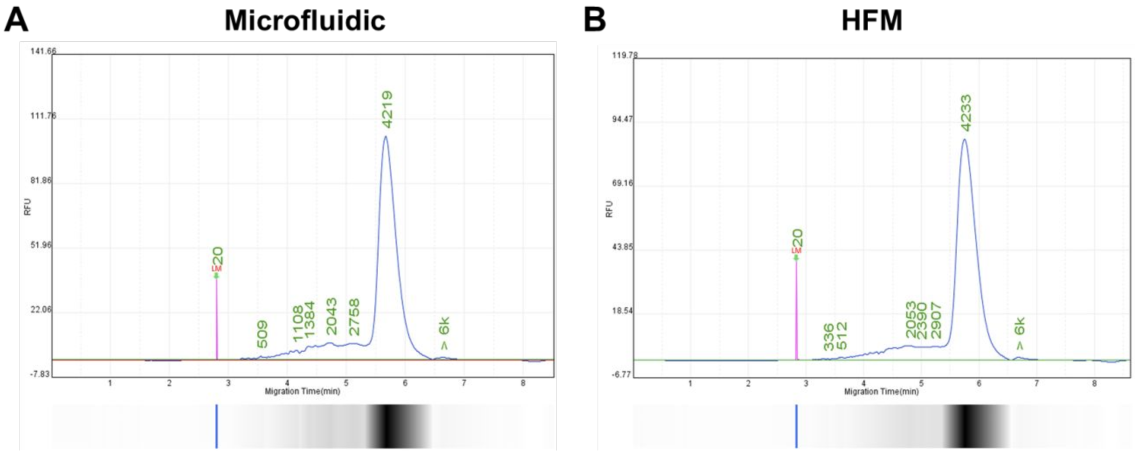

mRNA integrity analysis. (A) Spike mRNA extracted from LNPs prepared by microfluidics was analyzed by CE. **(B)** Spike mRNA extracted from LNPs prepared by HFM was analyzed by CE.

Fig. S20.

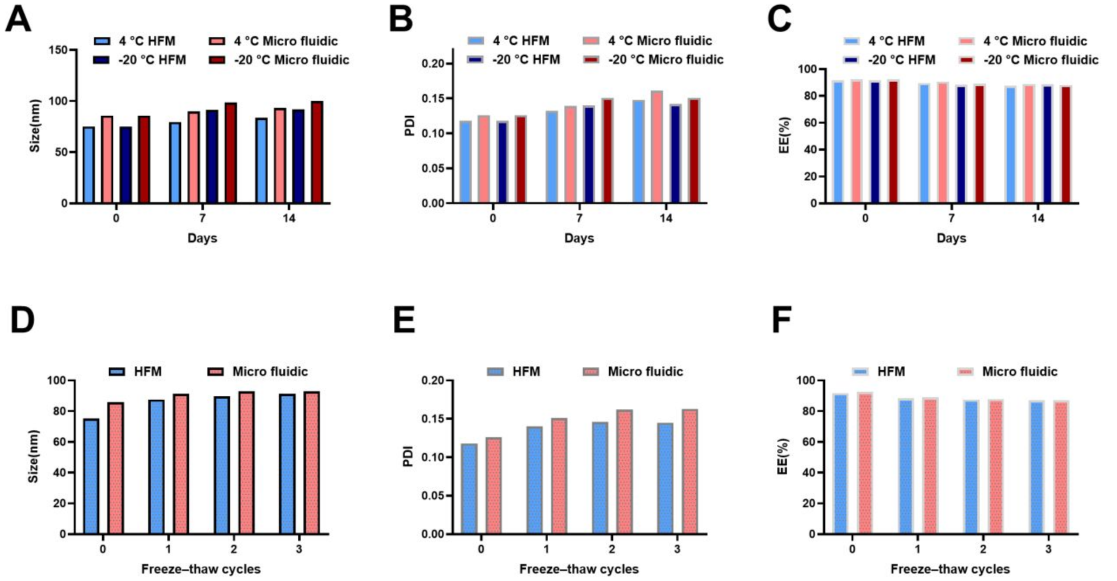

LNP stability analysis. **(A)** Particle size, **(B)** polydispersity index (PDI), and **(C)** encapsulation efficiency (EE) of LNPs prepared via the HFM platform and conventional microfluidic systems under different storage conditions. LNPs were stored at 4 °C for stability assessment (Day 0, 7, and 14), or at −20 °C in the presence of 10% (w/v) sucrose as a cryoprotectant. **(D)** Particle size, **(E)** PDI, and **(F)** EE of LNPs prepared by different methods with different freeze-thaw cycles.

Fig. S21.

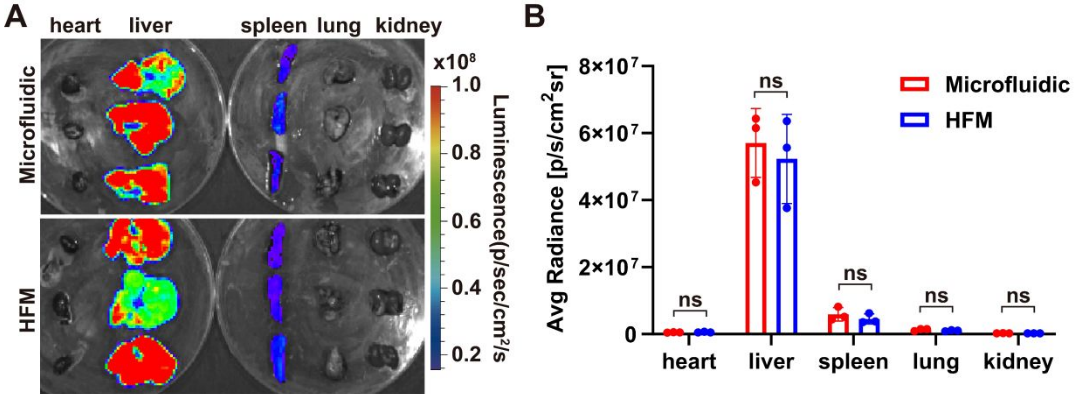

Biodistribution of LNPs prepared by microfluidic and HFM methods. (A) IVIS images of major organs 6 h post-treatment. **(B)** Quantification of average bioluminescence intensity based on A.

Fig. S22.

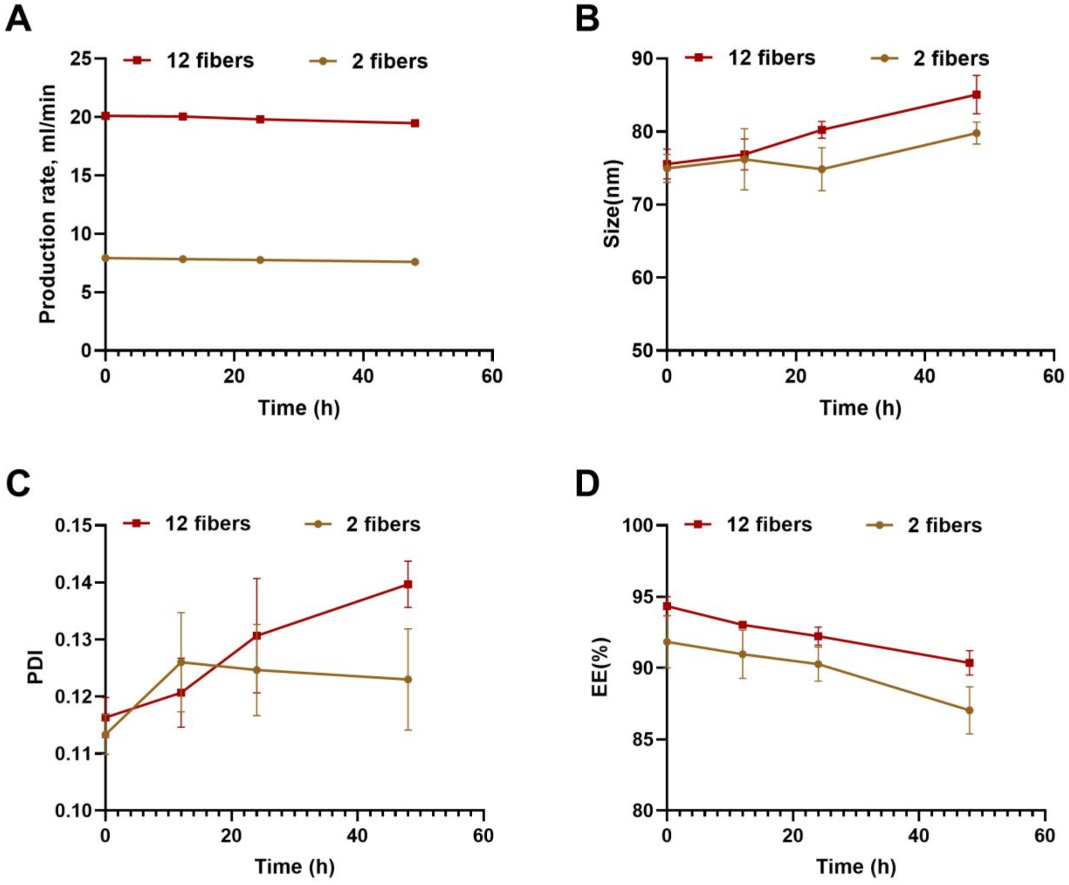

Long-term continuous operation analysis. (A) Long-term production rate analysis. **(B)** Long-term LNP size, **(C)** PDI and **(D)** EE% analysis.

Fig. S23.

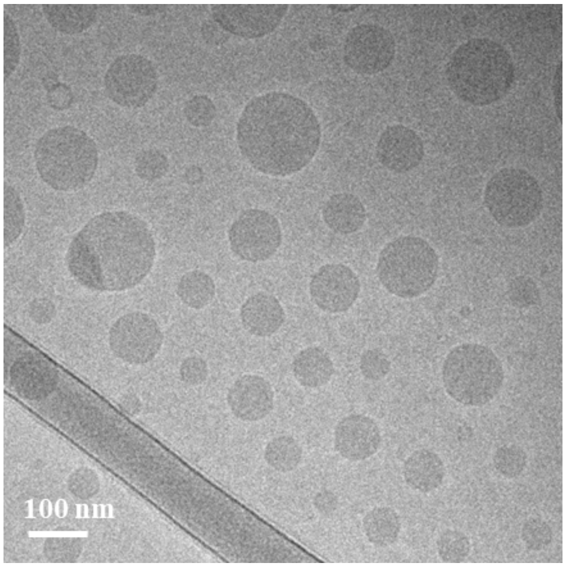

Cryo-TEM demonstrates uniform particle size and morphology of LNP's produced via microfluidic method. Scale bar = 100 nm

Fig. S24.

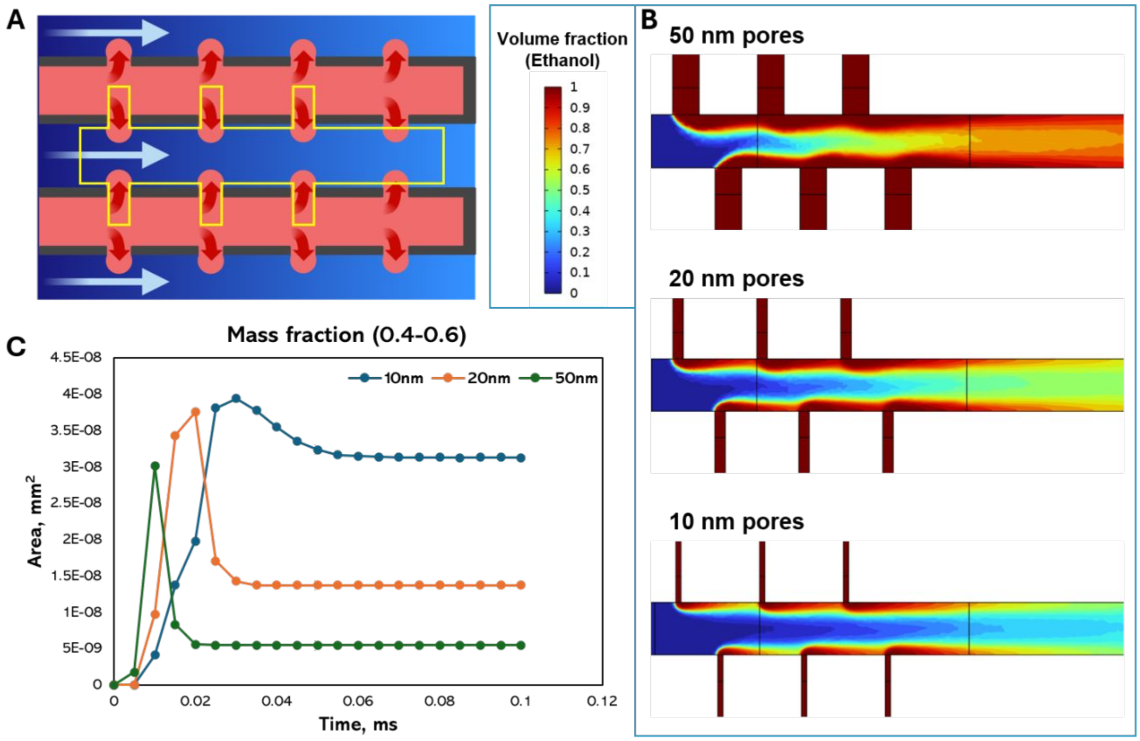

Mass-fraction-based simulation of nanopore-mediated mixing in the hollow fiber membrane system. (A) Schematic illustration of the hollow fiber membrane configuration, where the aqueous phase flows axially through the lumen and the ethanolic lipid/organic phase is injected radially through membrane nanopores. The yellow box indicates the simplified two-dimensional pore–channel domain used for simulation. **(B)** Simulated organic-phase mass-fraction distributions under different pore-size conditions. The color scale ranges from 0 to 1, corresponding to aqueous-rich and organic-rich regions, respectively. **(C)** Contact area analysis of models with different membrane pore sizes.

Computational Modeling with Miscible Mixing Model

Computational simulations were performed using COMSOL Multiphysics to investigate the early-stage mixing behavior within the hollow fiber membrane (HFM) system. A two-dimensional (2D) pore-channel model was constructed to represent the nanoporous membrane architecture as an array of uniformly distributed pore openings with pore diameters ranging from 5 to 200 nm. The model consisted of a central aqueous-flow channel and multiple pore inlets through which the organic phase was introduced, mimicking transmembrane injection from the shell side into the lumen-side flow.

The simulations were performed under isothermal conditions at 293.15 K using the Laminar Flow and Transport of Concentrated Species interfaces coupled through the Reacting Flow multiphysics module. The model was designed to characterize the transient evolution of solvent mixing immediately following nanopore injection.

Governing Equations

The flow field was described by the transient continuity and momentum equations:

$$\frac{\partial \rho}{\partial t} + \nabla \cdot (\rho \mathbf{u}) = 0$$

$$\rho \left(\frac{\partial \mathbf{u}}{\partial t} + \mathbf{u} \cdot \nabla \mathbf{u} \right) = -\nabla p + \nabla \cdot \boldsymbol{\tau}$$

where

- ρ is the mixture density,
- $\mathbf{u}$ is the velocity vector,
- p is the pressure,
- $\boldsymbol{\tau}$ is the viscous stress tensor.

The viscous stress tensor was defined using the Newtonian constitutive relation:

$\boldsymbol{\tau} = \mu(\nabla \mathbf{u} + (\nabla \mathbf{u})^T)$where μdenotes the dynamic viscosity of the fluid mixture.

Because the characteristic pore dimensions were on the nanometer scale and the Reynolds number was substantially below unity ($Re \ll 1$), all simulations were performed under laminar flow conditions without the use of a turbulence model.

Species Transport

Mixing between water and ethanol was modeled using the Transport of Concentrated Species interface based on the Maxwell–Stefan formulation. The transient mass-fraction equation for ethanol was solved as:

$$\frac{\partial(\rho w_2)}{\partial t} + \nabla \cdot (\rho \mathbf{u} w_2) = \nabla \cdot (\rho D \nabla w_2)$$ where

- w_2 is the ethanol mass fraction,
- D is the binary diffusion coefficient of the water–ethanol system,
- ρ is the local mixture density.

The binary diffusion coefficient was specified as:

$D = 1.2 \times 10^{-9}\ \mathrm{m^2 \cdot s^{-1}}$ consistent with reported values for water–ethanol mixtures.

Mixture Density and Viscosity

The local fluid density was calculated as a concentration-dependent mixture property using the reciprocal mixing rule:

$$\frac{1}{\rho} = \frac{w_2}{\rho_E} + \frac{1 - w_2}{\rho_W}$$ where

- $\rho_E = 789\ \mathrm{kg \cdot m^{-3}}$ is the density of ethanol,
- $\rho_W = 998\ \mathrm{kg \cdot m^{-3}}$ is the density of water.

Similarly, the dynamic viscosity was treated as a concentration-dependent mixture property:

$$\frac{1}{\mu} = \frac{w_2}{\mu_E} + \frac{1 - w_2}{\mu_W}$$ where

- $\mu_E = 1.074 \times 10^{-3}$ Pa·s is the viscosity of ethanol,
- $\mu_W = 1.002 \times 10^{-3}$ Pa·s is the viscosity of water.

Boundary Conditions

The aqueous phase was introduced through the main channel inlet, while the ethanol phase was injected through nanopore openings representing transmembrane transport. At the aqueous inlet, the ethanol mass fraction was specified as:

$w_2 = 0$ and at the pore inlets:

$w_2 = 1$ corresponding to pure water and pure ethanol, respectively.

Velocity boundary conditions were applied at all inlets, a constant-pressure condition was imposed at the outlet, and no-slip boundary conditions were enforced on all solid walls. Gravitational effects were neglected.

Numerical Implementation and Data Analysis

Transient simulations were performed using a time-dependent solver with a time step of 1×10^{-4}s. The computational domain was discretized using a physics-controlled mesh consisting of approximately 17,000–20,000 triangular elements. Mesh independence was verified by comparing the velocity and concentration fields across multiple mesh densities.

The spatial distribution of ethanol was quantified using the ethanol mass-fraction field (w_2). To evaluate the extent of local mixing, an interaction region was defined as the transitional concentration zone:

$0.4 < w_2 < 0.6$which represents regions where water and ethanol coexist in comparable proportions. The area of this interaction region was calculated using surface integration and subsequently normalized to obtain the relative contact surface used for comparison among different pore sizes.

Table S1.

Buffer Strength	10	25	50	150	250
**pH=3**	82.8±6.0	90.7±6.6	82.8±6.0	117.2±8.5	192.0±13.9
**pH=4**	126.4±9.2	93.8±6.8	109.8±8.0	133.9±9.7	234.7±17.0
**pH=5**	128.1±9.3	117.4±8.5	130.8±9.5	144.0±10.4	211.8±15.4
**pH=6**	128.6±9.3	123.6±9.0	130.4±9.5	159.6±11.6	238.0±17.3
**pH=7**	192.6±14.0	181.9±13.2	175.0±12.7	248.8±18.0	286.2±20.8
**pH=8**	405.2±29.4	346.6±25.1	295.2±21.4	322.9±23.4	360.5±26.1

Data set for heat map of particle size as a function of buffer strength. (mean ± standard deviation, n=3)

Table S2.

Buffer Strength	10	25	50	150	250
**pH=3**	0.23±0.04	0.20±0.08	0.12±0.03	0.12±0.02	0.21±0.04
**pH=4**	0.22±0.07	0.17±0.02	0.15±0.04	0.13±0.02	0.25±0.03
**pH=5**	0.25±0.06	0.18±0.04	0.18±0.04	0.14±0.03	0.27±0.05
**pH=6**	0.29±0.05	0.21±0.05	0.22±0.04	0.27±0.05	0.30±0.07
**pH=7**	0.25±0.05	0.24±0.04	0.26±0.05	0.30±0.09	0.30±0.08
**pH=8**	0.47±0.08	0.37±0.09	0.42±0.10	0.56±0.12	0.61±0.15

Data set for heat map of PDI as a function of buffer strength. (mean ± standard deviation, n=3)

Table S3.

Buffer Strength	10	25	50	150	250
**pH=3**	95.7±3.4	92.4±1.7	95.0±4.1	79.1±4.0	63.3±4.4
**pH=4**	97.7±3.1	97.4±2.1	96.1±3.1	77.4±4.6	67.5±2.2
**pH=5**	92.0±1.5	91.6±4.9	91.1±4.9	76.4±5.5	66.4±5.5
**pH=6**	87.3±3.8	91.2±4.9	87.2±4.7	72.0±3.8	64.9±4.4
**pH=7**	80.0±3.7	85.6±4.0	79.9±3.7	70.1±5.2	60.1±6.1
**pH=8**	33.4±9.3	35.5±9.8	31.2±8.7	30.2±8.4	25.1±10.0

Data set for heat map of EE% as a function of buffer strength. (mean ± standard deviation, n=3)

Table S4.

Mixing Platform	Characteristic Mixing Time (τ_mix)	Peclet Number (Pe)	Typical Throughput
**T-junction microfluidics**	1–10 ms	10^2–10^3	1–50 mL/min
**Staggered herringbone mixer (SHM)**	0.5–5 ms	10^2–10^4	10–100 mL/min
**Confined impingement jet (CIJ)**	0.1–1 ms	10^3–10^5	50–500 mL/min
**Nanopore-mediated HFM**	0.1–2 ms	10^4–10^6 (local pore scale)	Scalable (>100 mL/min to L/min, theoretical)

The characteristic mixing time (τ_mix) was estimated based on literature-reported values for microfluidic systems and scaling analysis for nanopore-mediated injection. The Peclet number (Pe = UL/D) reflects the relative contribution of convection versus diffusion, where higher values indicate convection-dominated transport. Notably, the HFM platform operates at significantly reduced mixing length scales (nanometer-scale pores), leading to rapid local dilution and enhanced mixing efficiency despite high Pe conditions.
